# Association between adding salt in food and dementia in European descent: A mendelian randomization study

**DOI:** 10.1002/brb3.3516

**Published:** 2024-05-03

**Authors:** Ren Zhou, Fei Chen, Lei Zhang, Yu Sun, Rong Hu, Jia Yan, Hong Jiang

**Affiliations:** ^1^ Department of Anesthesiology The Ninth People's Hospital of Shanghai Jiao Tong University School of Medicine Shanghai P. R. China

**Keywords:** Alzheimer's disease, dementia, parkinson's disease, salt intake

## Abstract

**Background:**

High salt intake has been proposed as a risk factor for dementia. However, causal relationship between salt intake and dementia remains uncertain.

**Purpose:**

The aim of this study was to employ a mendelian randomization (MR) design to investigate the causal impact of salt intake on the risk of dementia.

**Methods:**

Genome‐wide association study (GWAS) data of exposures and outcomes (any dementia, cognitive performance, different types of dementia, Alzheimer's disease [AD], and Parkinson's disease) were obtained from the IEU database. MR estimates were generated though inverse‐variance weighted model. MR‐Egger, weighted median, and MR‐Pleiotropy Residual Sum and Outlier (MR‐PRESSO) method also used in our study. Sensitivity analyses included Cochran's *Q* test, MR‐Egger intercept, MR‐PRESSO global test and outlier test, leave‐one‐out analysis, and funnel plot assessment.

**Results:**

Our MR analysis provided evidence of a causal association between high salt added to food and dementia (odds ratio [OR] = 1.73, 95% confidence interval [CI]: 1.21–2.49, and *p* = .003), dementia in AD (OR = 2.10, 95% CI: 1.15–3.83, and *p* = .015), and undefined dementia (OR = 2.61, 95% CI: 1.26–5.39, and *p* = .009). Higher salt added was also associated with increased risk of AD (OR = 1.80, 95% CI: 1.12–2.87, and *p* = .014) and lower cognitive performance (*β* = −.133, 95% CI: −.229 to −.038, and *p* = .006).

**Conclusion:**

This study provides evidence suggesting that high salt intake is causally associated with an increased risk of developing dementia, including AD and undefined dementia, highlighting the potential importance of reducing salt consumption as a preventive measure.

## Graphical abstract

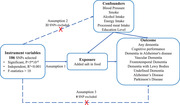

## 1 INTRODUCTION

Dementia is a complex and progressive neurodegenerative disorder characterized by a decline in cognitive function, memory loss, and impaired daily functioning. It is a major global health issue affecting millions of individuals, their families, and caregivers (Gale et al., 2018). In 2023, more than 55 million people had dementia worldwide. Every year, there are nearly 10 million new cases. The most common form of dementia is Alzheimer's disease (AD), accounting for an estimated 60%–80% of cases. Other types, such as vascular dementia (VaD), frontotemporal dementia (FTD), and dementia with Lewy bodies (DLB), also contribute to the overall prevalence (Aarsland, 2020; Rossor et al., 2010). The exact causes of dementia are not fully understood, but a combination of genetic, environmental, and lifestyle factors is believed to play a role. Genetic predispositions, including the APOE ε4 (Serrano‐Pozo et al., 2021), can elevate susceptibility. Environmental exposure may contribute to cognitive decline (Younan et al., 2022). Lifestyle choices, including physical activity, diet, and cognitive stimulation, play pivotal roles in preventing dementia (Di Marco et al., 2014). Moreover, structural determinants, including access to healthcare and neighborhood environments, and social determinants, including education and social support networks, profoundly impact dementia risk (Adkins‐Jackson et al., 2023). Understanding risk factors associated with dementia is crucial for developing effective prevention strategies and interventions (Baumgart et al., 2015). Dietary prevention strategies represent vital aspects of dementia risk reduction. Various dietary patterns and interventions have been explored, offering insights into their potential efficacy. For instance, special dietary patterns such as mediterranean diet, which emphasizes consumption of fruits, vegetables, whole grains, fish, and healthy fats while limiting the intake of red meat, processed foods, and saturated fats, have shown a significant protective effect on dementia (Chen et al., 2023; Song et al., 2024). Additionally, studies have investigated the impact of diets rich in antioxidants, omega‐3 fatty acids, and certain vitamins and minerals on cognitive health. The role of calorie restriction and intermittent fasting in promoting brain function and reducing the risk of dementia has garnered attention (Solfrizzi et al., 2017).

High salt intake is a prevalent dietary habit worldwide, contributing to various health concerns. A review in 2022 indicates that most countries in the WHO European Region reported salt intake above WHO recommended maximum levels, ranging from 4.27 to 18.51 g/day (Kwong et al., 2023). This excessive intake is often fueled by a combination of cultural preferences, food processing practices, and omnipresence of salt in numerous food products. In recent years, increased intakes of processed and restaurant foods have been accompanied by increased salt intake. High salt intake has emerged as a potential modifiable risk factor for the development and progression of many disease (Strazzullo et al., 2009). Studies have suggested that a high‐salt diet may contribute to hypertension (Rust & Ekmekcioglu, 2017), a known risk factor for VaD. Elevated blood pressure can damage blood vessels in the brain, leading to reduced blood flow and an increased risk of cognitive impairment (Coca, 2013). Additionally, previous study found high salt intake may directly impact the brain by tau protein and lead to dementia, including AD, in animal model (Faraco et al., 2019). A cohort study conducted in China with elderly individuals has further substantiated the link between dietary salt intake and dementia (Liu et al., 2023). However, more research is needed to establish a definitive causal relationship between high salt intake and dementia and to better understand the underlying mechanisms involved.

Adding salt to food, typically done at the table, is a widespread eating habit that is strongly connected to an individual's enduring fondness for salty flavors and their habitual salt consumption (Quader et al., 2019; Van der Veen et al., 1999). Research has revealed that the frequency of adding salt to foods can serve as an indicator of a person's long‐standing preference for salty tastes and their overall sodium intake and have a direct association with 24‐h urinary sodium (Ma et al., 2022). Therefore, incorporating salt into meals offers a distinctive means to assess the relationship between habitual sodium consumption and individuals’ choices.

Mendelian randomization (MR) is an innovative study design that uses genetic variants as instrumental variables (IVs) to investigate causal relationships between exposures and outcomes (Katikireddi et al., 2018). Randomized controlled trial (RCT) is the gold standard method of inferring causality. However, there may be ethical and financial reasons as to why RCTs are not a viable means of determining causality (Adkins‐Jackson et al., 2023). MR is an epidemiological technique that has been developed as a means of not only avoiding the pit falls classically associated with RCTs, such as confounding variables, but also examining causal factors for phenotypes that would not be appropriate for RCTs. MR leverages naturally occurring genetic variations associated with modifiable risk factors to provide insights into potential causal links while minimizing the confounding and reverse causation issues that commonly affect observational studies. In the context of high salt intake and dementia, MR studies offer a unique opportunity to explore whether there is a causal relationship between these factors. Studies have been conducted using MR methods to assess the health effects of salt added to food and risk factors for dementia (Jin et al., 2022; Sproviero et al., 2021; Wang et al., 2023; Zhou et al., 2021). These studies used inverse‐variance weighted (IVW) as the main MR method and found that salt intake can affect atrial fibrillation and BMI; the effects of hypertension, systemic lupus erythematosus, and so on on dementia were also noted. The application of MR in studying the relationship between high salt intake and dementia holds promise in advancing our understanding of this complex association and informing preventive strategies and interventions.

In this present study, MR was employed to examine the potential correlation between salt consumption in food and an elevated risk of dementia, including AD and Parkinson's disease (PD).

## 2 METHODS

### 2.1 Exposure data sources

All genome‐wide association study (GWAS) data in our study are from the IEU OpenGWAS database. The genetic variants associated with salt added to food were extracted from the UK Biobank, which had a dataset identifier of ukb‐b‐8,121. This dataset consisted of 462,630 individuals of European ancestry. Information on the frequency of adding salt to foods was collected using a touchscreen questionnaire. Participants were asked, “Do you add salt to your foods? (Do not include salt used in cooking),” and they selected 1 answer from 5 options: never/rarely; sometimes; usually; always; or prefer not to answer. Those who preferred not to answer were considered to have missing information and were excluded from the analysis. GWAS sample information is presented in Table 1.

**TABLE 1:** Exposure and oucomes genome‐wide association study (GWAS) data details.

Type	Item (GWAS ID)	Consortium	Category	Sample size	SNPs number	Ethnicity	Year
Exposure	Dietary salt intake (ukb‐b‐8,121)	MRC‐IEU	Categorical (single)	462,630	9851,867	European	2018
Outcomes	Any dementia (finn‐b‐F5_DEMENTIA)	FinnGen	Binary	216,771 7284 cases and 209,487 controls	16,380,463	European	2021
	Cognitive performance (ebi‐a‐GCST006572)	NA	Continuous (standardized score)	257,841	10,066,414	European	2018
	Dementia with Lewy bodies (ebi‐a‐GCST90001390)	NA	Binary	6618 2591 cases and 4027 controls	7593,175	European	2021
	Frontotemporal dementia (ieu‐b‐43)	NA	Binary	3024 515 case and 2509 controls	494,577	European	2010
	Dementia in Alzheimer's disease (finn‐b‐F5_ALZHDEMENT)	FinnGen	Binary	211,678 2191 cases and 209,487 controls	16,380,451	European	2021
	Vascular dementia (finn‐b‐F5_VASCDEM)	FinnGen	Binary	212,389 881 cases and 211,508 controls	16,380,457	European	2021
	Undefined dementia (finn‐b‐F5_Dementia_U)	FinnGen	Binary	215,511 1589 cases and 213,922 controls	16,380,464	European	2021
	Alzheimer's disease (finn‐b‐G6_ALZHEIMER)	FinnGen	Binary	218,792 3899 cases and 214,893 controls	16,380,466	European	2021
	Parkinson's disease (finn‐b‐G6_PARKINSON)	FinnGen	Binary	218,792 2162 cases and 216,630 controls	16,380,466	European	2021

Abbreviation: SNPs, single‐nucleotide polymorphisms.

### 2.2 Outcome data sources

Regarding the GWAS data for dementia, we also obtained it from the IEU OpenGWAS database.

The primary outcome in our study is the dementia (any type), we selected data from the finn consortium (ID: finn‐b‐F5_DEMENTIA), which included 7284 cases and 209,487 controls of European ancestry.

Moreover, cognitive performance and different types of dementia were also evaluated in our study. GWAS data of cognitive performance were from Lee'S study with a dataset identifier of ebi‐a‐GCST006572 (Lee et al., 2018). For different types of dementia, the summary‐level GWAS data are mainly from Finngen database. Data for VaD with the dataset identifier finn‐b‐F5 VASCDEM. Data for dementia in AD were identifier of finn‐b‐F5_ALZHDEMENT. Data for undefined dementia were identifier of finn‐b‐F5_Dementia_U. The GWAS data for DLB were derived from an independent multicenter study with a dataset identifier of ebi‐a‐GCST90001390 (Chia et al., 2021). The summary statistics data of FTD from the dataset ieu‐b‐43 were also derived from an independent study (Van Deerlin et al., 2010).

To further clarify our results, we also included GWAS data on AD and PD. For our investigation into AD, we obtained GWAS summary data from the European population, which had a dataset identifier of finn‐b‐G6_ALZHEIMER. For our investigation into PD, we obtained a dataset identifier of finn‐b‐G6_PARKINSON. Both those GWAS summary data from the European population. GWAS sample information is presented in Table 1.

For GWAS data from Finngen database(any dementia, VaD, dementia in AD, undefined dementia, AD, and PD), different types of dementia identified according to the International Classification of Disease‐10 criteria (https://risteys.finregistry.fi/). For FTD, individuals of European descent with dementia clinically +/− motor neuron disease and an autopsy diagnosis of FTD‐TDP confirmed by TDP‐43 IHC were included as case (Van Deerlin et al., 2010). For DLB, patients were diagnosed with pathologically definite or clinically probable disease according to consensus criteria (Chia et al., 2021). Specific information for each type of dementia is shown in Table S1.

For cognitive performance, the GWAS data were combined data from COGENT and UKB. In UKB, a test contains 13 logic and reasoning questions with a 2‐min time limit and was designed as a measure of fluid intelligence. In COGENT, cognitive performance was summarized from 35 component studies; the phenotype used was the first unrotated principal component of performance on at least 3 neuropsychological tests (or at least two IQ‐test subscales). Across the individual studies, the first PC explained an average of 41% of the variance in test performance. The detailed methodology can be found in the original research (Lee et al., 2018).

Both exposure and outcome GWAS data have already been adjusted for sex and age.

### 2.3 Selection of instrumental variables

**FIGURE 1:**
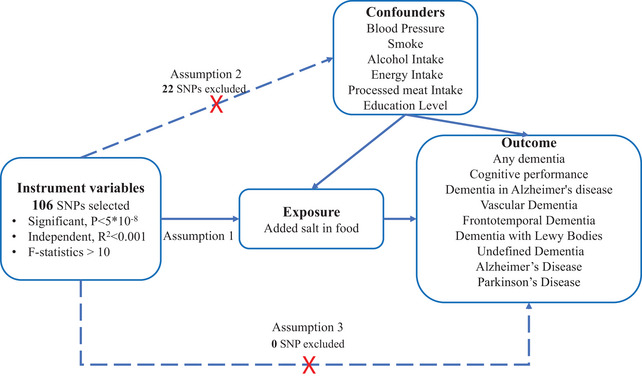
Directed acyclic graph is used to illustrate the hypothesis of added salt in food impact on dementia. The presence of a dotted line indicates a potential direct causal relationship or pleiotropic effects between the exposure (added salt in food) and the outcome (dementia).

We employed a two‐sample MR design to examine the causal impact of added salt in food on dementia, as depicted in Figure 1. The MR design hinges on three fundamental assumptions. First, the genetic IVs must exhibit a strong correlation with salt intake (Assumption 1). Second, confounding factors should not influence the chosen IVs, thereby avoiding any potential distortion in the relationship between salt intake and dementia (Assumption 2). Lastly, the IVs can only influence the risk of dementia through salt intake (Assumption 3) (Didelez & Sheehan, 2007).

Based on those assumption, we employed five criteria to screen the single‐nucleotide polymorphisms (SNPs) used as IVs: (1) SNPs with a significant correlation (*p* < 5 × 10^−8^) with salt intake were selected, ensuring their independence from each other (*r* ^2^ < .001) within a clumping distance of 10,000 kb; (2) SNPs associated with the outcomes of interest (*p* < 5 × 10^−8^) were excluded from the IVs; (3) To mitigate the influence of potential confounding factors, SNPs associated with blood pressure, smoking, energy intake, education level, Processed meat intake, and alcohol consumption were excluded. This information was obtained from the PhenoScanner database V2 (http://www.phenoscanner.medschl.cam.ac.uk/, accessed on May 30 2023); (4) To ensure a strong correlation between the IVs and the exposure factors, we calculated the *F*‐statistic. The *F*‐statistic was derived using the formula: $F\;=\frac{R^{2}\times \left(N-K-1\right)}{K\times (1-R^{2})}\;$, where *R* ^2^ represents the coefficient of determination, *N* is the sample size, and *K* is the number of IVs; (5) To maintain consistency between the effects of SNPs on exposures and outcomes, palindromic SNPs with intermediate allele frequencies were excluded from the analysis. By implementing these criteria, we aimed to establish robust IVs that would enable us to examine the potential causal associations between salt intake and dementia outcomes. The details of selected SNPs are shown in Table S1.

### 2.4 Statistical analysis

In our study, the IVW method played a crucial role in conducting the major MR analysis. This approach combines the Wald ratio of individual SNPs. It assumes that IVs only affect outcomes through specific exposures. When there is no horizontal pleiotropy, the IVW method can provide most credibly results (Burgess et al., 2013). In MR study, IVW commonly used as the major method.

To ensure the stability of our findings, several complementary analyses were performed. These included the MR‐Egger (Bowden et al., 2015), weighted median (WM), and MR pleiotropy residual sum and outlier test (MR‐PRESSO) (Verbanck et al., 2018) methods in subsequent sensitivity analyses. Cochran's *Q* test was employed to evaluate potential heterogeneity and horizontal pleiotropy. Furthermore, the MR‐PRESSO analysis was used to identify and eliminate outliers and moderate horizontal pleiotropy. In addition, the sensitivity analysis of this study comprised the following components for our primary outcome: First, in addition to the exclusion of confounding factor‐related SNPs, adjustment for confounders was performed by multivariable MR (MVMR) in the primary outcome; second, SNPs associated with BMI were additionally excluded from the primary outcome, even though previous studies have revealed a causal relationship between salt intake and BMI, and BMI is more feasible mediator in this study. To assess the impact of individual variants on the overall results, a leave‐one‐out analysis was performed. False discovery rate was calculated for multiple corrections.

All statistical analyses were performed using R software (version 4.0.5) with the TwoSampleMR package (Hemani et al., 2018), as well as MR‐PRESSO. For the original version, data were analyzed from May to June 2023.

## 3 RESULTS

### 3.1 Selection of instrumental variables

Through screening for SNPs associated with exposures (*p* < 5 × 10^−8^) and removing the linkage disequilibrium, 106 independent SNPs were selected for exposure. After excluding 22 SNPs associated with confounding factors, we used the remaining 84 SNPs as IVs. No SNP removed due to associated with outcome variables or *F*‐statistics value <10 Table S2). For outcome cognitive performance, VaD, AD, PD, dementia in AD, undefined dementia, and any types of dementia, 5 SNPs were removed for palindromic and incompatible alleles (rs13084934, rs6443950, rs9375448, rs976179, and rs55897719). For other outcomes (DLB and FTD), 3 SNPs (rs13084934, rs9375448, and rs976179) removed.

### 3.2 Results of MR

**FIGURE 2:**
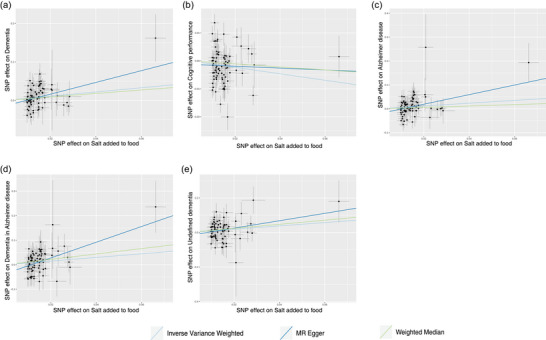
Scatter plot depicts the results of mendelian randomization (MR) analyses investigating the association between dietary salt intake and dementia. Each line in the plot represents a different MR method, and the slope of each line represents the estimated association between the two variables: (a) scatter plot between added salt in food and any dementia; (b) scatter plot between added salt in food and cognitive performance; (c) scatter plot between added salt in food and Alzheimer's disease; (d) scatter plot between added salt in food and dementia in Alzheimer's disease; (e) scatter plot between added salt in food and undefined dementia.

The results revealed a significant causal relationship between salt intake and the risk of any dementia (odds ratio [OR] = 1.73, 95% confidence interval [CI]: 1.21–2.49, and *p* = .003) in IVW methods. MR‐Egger, MR‐PRESSO, and WM analyses also showed a similar trend, and the association was significant in MR‐Egger analysis (OR = 4.63, 95% CI: 1.41–15.22, and *p* = .014) and MR‐PRESSO analysis (OR = 1.70, 95% CI: 1.20–2.41, and *p* = .004) (Table 2 and Figure 2).

**TABLE 2:** Mendelian randomization (MR) results and sensitivity analysis for association of adding salt in food and dementia risk.

Outcome (GWAS ID)	Method	Number of SNP	OR or β (95% CI)	*p*	FDR
Any dementia (finn‐b‐F5_DEMENTIA)	MR Egger	78	4.63 (1.41, 15.22)	.014	0.042
	Weighted median	78	1.57 (.93, 2.67)	.093	0.214
	Inverse variance weighted	78	1.73 (1.21, 2.49)	.003	0.027
	PRESSO	78	1.70 (1.20, 2.41)	.004	0.036
Cognitive performance(ebi‐a‐GCST006572)	MR Egger	79	−.08 (−.392,.232)	.618	0.729
	Weighted median	79	−.073 (−.158,.013)	.095	0.214
	Inverse variance weighted	79	−.133 (−.229, −.038)	.006	0.027
	PRESSO	79	−.117 (−.212, −.021)	.019	0.043
Dementia with Lewy bodies (ebi‐a‐GCST90001390)	MR Egger	74	.74 (.07, 7.33)	.796	0.796
	Weighted median	74	.95 (.33, 2.68)	.919	0.919
	Inverse variance weighted	74	.99 (.49, 2.01)	.983	0.983
	PRESSO	74	.95 (.48, 1.89)	.885	0.885
Dementia in Alzheimer's disease (finn‐b‐F5_ALZHDEMENT)	MR Egger	78	24.66 (3.35, 181.41)	.002	0.018
	Weighted median	78	3.00 (1.24, 7.27)	.015	0.108
	Inverse variance weighted	78	2.10 (1.15, 3.83)	.015	0.027
	PRESSO	78	2.09 (1.19, 3.68)	.012	0.043
Vascular dementia (finn‐b‐F5_VASCDEM)	MR Egger	78	2.60 (.10, 68.85)	.570	0.729
	Weighted median	78	1.70 (.44, 6.63)	.442	0.600
	Inverse variance weighted	78	2.35 (.89, 6.23)	.086	0.129
	PRESSO	78	1.96 (.74, 5.18)	.180	0.270
Frontotemporal dementia (ieu‐b‐43)	MR Egger	34	.22 (.00, 139.41)	.648	0.729
	Weighted median	34	2.63 (.19, 35.67)	.467	0.600
	Inverse variance weighted	34	.73 (.11, 4.70)	.737	0.829
	PRESSO	34	.66 (.11, 3.80)	.646	0.727
Undefined dementia (finn‐b‐F5_Dementia_U)	MR Egger	78	8.51 (.76, 95.9)	.087	0.196
	Weighted median	78	3.28 (1.17, 9.24)	.024	0.108
	Inverse variance weighted	78	2.61 (1.26, 5.39)	.009	0.027
	PRESSO	78	2.22 (1.06, 4.64)	.038	0.068
Alzheimer's disease (finn‐b‐G6_ALZHEIMER)	MR Egger	78	7.46 (1.6, 34.72)	.012	0.042
	Weighted median	78	1.34 (.67, 2.66)	.404	0.600
	Inverse variance weighted	78	1.80 (1.12, 2.87)	.014	0.027
	PRESSO	78	1.78 (1.13, 2.80)	.015	0.043
Parkinson's disease (finn‐b‐G6_PARKINSON)	MR Egger	78	2.08 (.27, 15.95)	.485	0.729
	Weighted median	78	1.20 (.51, 2.84)	.677	0.762
	Inverse variance weighted	78	1.33 (.73, 2.45)	.355	0.456
	PRESSO	78	1.38 (.77, 2.48)	.281	0.361

Abbreviations: CI, confidence interval; GWAS, genome‐wide association study; OR, odds ratio; PRESSO, pleiotropy residual sum and outlier; SNP, single‐nucleotide polymorphism.

The results revealed a significant relationship between salt intake and the lower cognitive performance (*β* = −.133, 95% CI: −.229 to −.038, and *p* = .006), risk of dementia in AD (OR = 2.10, 95% CI: 1.15–3.83, and *p* = .015), and undefined dementia (OR = 2.61, 95% CI: 1.26–5.39, and *p* = .009) in IVW method. MR‐Egger, MR‐PRESSO, and WM analyses also showed a similar trend. For dementia in AD, all four MR method shown a significant association. For cognitive performance, only PRESSO analysis shown a significant association (*β* = −.117, 95% CI: −.221 to −.021, and *p* = .019). For undefined dementia, only WM analysis shown a significant association (OR = 3.28, 95% CI: 1.17–9.24, and *p* = .024) (Table 2 and Figure 2). For VaD, FTD, and DLB, no genetic association with salt intake was found using the IVW, MR‐Egger, WM, and MR‐PRESSO models (Table 2 and Figure S1).

Our results revealed an association between salt intake and the risk of AD (OR = 1.80, 95% CI: 1.12–2.87, and *p* = .014). This finding was consistent with the results obtained from MR‐Egger analysis (OR = 7.46, 95% CI: 1.60–34.72, and *p* = .012) and MR‐PRESSO (OR = 1.78, 95% CI: 1.13–2.80, and *p* = .015) analysis but only shown a trend with the WM analysis (Table 2 and Figure 2). Our analysis did not reveal any evidence of a potential causal association between salt intake and the risk of PD in the databases investigated. Consistent results were obtained from the IVW, MR‐Egger, WM, and MR‐PRESSO analyses (Table 2 and Figure S1).

**FIGURE 3:**
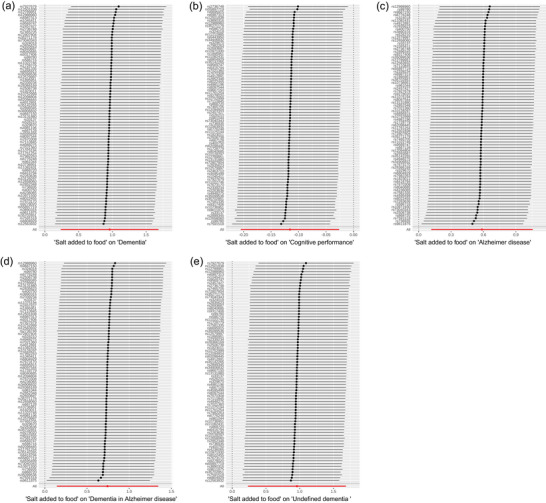
The figure displays the results of a leave‐one‐out analysis in mendelian randomization (MR). Each black line in the figure corresponds to the outcome of the MR analysis when one single nucleotide polymorphism (SNP) is removed from the analysis, whereas the remaining SNPs are used on the left: (a) leave‐one‐out analysis between added salt in food and any dementia; (b) leave‐one‐out analysis between added salt in food and cognitive performance; (c) leave‐one‐out analysis between added salt in food and Alzheimer's disease; (d) leave‐one‐out analysis between added salt in food and dementia in Alzheimer's disease; (e) leave‐one‐out analysis between added salt in food and undefined dementia.

There was only significant heterogeneity observed on outcome cognitive performance (Table 3). However, based on the results of PRESSO outlier test, the outlier did not change our results (*p* = .890). Only evidence of horizontal pleiotropy was found in outcome “dementia in AD” (Table 3). Random effects IVW method was more suitable for SNPs that have horizontal pleiotropy. Therefore, for this outcome, we used the random effects IVW (shown as IVW). The leave‐one‐out test demonstrated the stability of our results (Figure 3 and Figure S2), and no outlier SNPs were identified in the MR‐PRESSO analysis (Table 3). Funnel plot also proved the credibility of our study in IVW method (Figure S3). The result of MVMR on any dementia is similar to univariable MR (Table S3). Only AD shown a different effect in MVMR. The results when BMI is considered a confounder factor are also similar (Table S4).

**TABLE 3:** Sensitivity analysis of the mendelian randomization (MR) analysis results of exposures and outcomes.

Outcome	Heterogeneity test (MR‐Egger)		Heterogeneity test (IVW)		PRESSO method		Pleiotropy test (MR‐Egger intercept test)	
	*Q*	*p*	*Q*	*p*	Distortion test *p*	Global *p*	*I*	*p*
Any type dementia	79.42	.372	82.43	.315	NA	.346	−0.015	.094
Cognitive performance	243.89	<.001	244.98	.000	.83	<.001	−0.001	.559
Dementia with Lewy bodies	77.92	.296	77.99	.323	NA	.375	0.004	.791
Dementia in Alzheimer's disease	66.33	.778	72.76	.616	NA	.677	−0.036	.013
Vascular dementia	90.64	.121	90.64	.137	NA	.095	−0.001	.950
Frontotemporal dementia	36.21	.279	36.37	.314	NA	.408	0.018	.706
Undefined dementia	86.20	.199	87.34	.197	NA	.082	−0.017	.320
Alzheimer's disease	76.49	.463	80.13	.381	NA	.442	−0.021	.061
Parkinson's disease	86.19	.199	86.41	.217	NA	.280	−0.007	.656

Abbreviations: IVW, inverse‐variance weighted; PRESSO, pleiotropy residual sum and outlier.

## 4 DISCUSSION

The present MR study aimed to explore the correlation between salt intake and the risk of dementia. With a focus on investigating the impact of salt intake on cognitive performance and different types of dementia, including dementia in AD, VaD, FTD, DLB, and undefined dementia. We also evaluated the association between salt intake with AD and PD. The results show the association between salt intake with dementia, cognitive performance, and a possible association with AD but not PD. The types of dementia association with salt intake are dementia in AD and undefined dementia. Our findings further proved the relationship between salt intake and cognitive health.

Numerous studies have highlighted the detrimental health effects of excessive salt intake, which have been extensively researched. Previous investigations have established that high salt intake can have negative impacts on cardiovascular and cerebrovascular functions, potentially leading to cognitive deficits and dementia (Perry, 2000). In 2019, a study directly observed that high salt intake could induce dementia in rats, providing early evidence of the association (Faraco et al., 2019). Subsequently, a recent study conducted on a cohort in China found that higher salt intake affected cognitive function and increased the incidence of dementia among older adults (Liu et al., 2023). Our study builds upon these existing findings provide evidence of a causal relationship between salt intake and the occurrence of dementia thought a MR method.

Furthermore, our study explored the role of salt intake in specific types of dementia. Although salt intake was found to have an impact on overall dementia risk, the association between salt intake and VaD was not significant. Hypertension, a known risk factor for VaD, has been extensively studied in relation to the effects of salt intake on blood pressure (He et al., 2020; Littlejohns et al., 2023). It is widely understood that long‐term uncontrolled hypertension can lead to damaged blood vessels in the brain and resulting in white matter damage, silent lacunae, and cortical disconnection (Nagai et al., 2010). In addition, previous studies have also found an association between hypertension and AD (Lennon et al., 2021). However, previous research both found that salt may lead dementia not though changed the blood pressure. Our study, along with previous research, found that the association between salt intake and dementia remained significant even after excluding genetic variants associated with hypertension (Faraco et al., 2019; Wang et al., 2023). Conversely, the association between salt intake and VaD was not significant, even when blood pressure‐related genetic variants were not excluded (data not shown). This suggests that the effect of salt on dementia may not solely rely on modulating blood pressure, or at least not entirely.

Previous studies have suggested that salt may primarily impact dementia through its influence on tau protein, possible mechanisms may be related to a deficiency of complex nitric oxide due to a high salt diet (Faraco et al., 2019). In healthy neurons, tau is found to carry multiple phosphate groups, primarily within its microtubule assembly domain. However, in AD and other tauopathies, there is a significant increase in tau phosphorylation and aggregation, which are widely recognized as pathological hallmarks. As a result, there has been a growing emphasis on investigating tau posttranslational modifications within the context of these diseases. Researchers are actively exploring the various modifications that occur to tau in order to better understand the underlying mechanisms and potentially develop therapeutic interventions (Wegmann et al., 2021). Our study found that high salt has a strong association with dementia caused by AD, which is consistent with previous studies. The higher salt intake was also increased the risk of AD. Notably, a previous study based on the UK Biobank database found an association between salt intake and fluid intelligence in a population with high‐risk AD (Klinedinst et al., 2020), which is consistent with our study. However, due to limitations in our data structure, we could not analyze the relationship between salt intake and AD specifically in high‐risk groups, such as the elderly. Further analysis is required to explore this aspect in more detail and gain a comprehensive understanding of the association.

Our study has clear advantages; first, despite the demonstrated association of salt with dementia and AD at the animal level, relevant studies in healthy populations are still scarce, and to our knowledge, only one observational study article from 2023 examined the relationship between salt and dementia in an elderly population (Liu et al., 2023). Our study further clarifies the causal relationship between salt and dementia through MR studies. Suggests an important role for salt intake control in dementia prevention and treatment. In addition, the GWAS selected for our primary positive outcome all contained larger samples (larger than hundred thousand), which enhances the credibility of our study. As with any research, certain limitations were also with our study. First, the reliance on self‐reported salt in food from a not‐validated dietary questionnaire introduced the potential for recall bias and measurement error. Although efforts were made to mitigate these limitations, it remains an inherent challenge in studies relying on self‐reported data. In addition, salt added is not directly equivalent to high salt consumption. High salt intake can also result from other food intake, including processed foods. This may also lead to bias. Moreover, other critical dietary risk factors for disease, including total caloric intake, fat intake, and potassium intake, were not recorded, which may also lead to bias. Second, we assumed a linear correlation between adding salt in food and the risk of dementia, whereas the actual relationship may be more complex. Salt intake also may interact with other factors in a nonlinear manner, requiring further investigation. Third, the sample size for certain types of dementia in our study was relatively small, potentially leading to false negative conclusions. Larger sample sizes would enhance the statistical power and reliability of the results. Fourth, although MR provides a useful method to infer causality by using genetic variants as proxies for exposures, it has limitations. These include assumptions like no pleiotropy, restricted to lifelong exposures, and potential underpowering for detecting small effects, which may impact the accuracy of causal inference. Finally, the absence of detailed demographic and clinical data for participants prevented subgroup analysis, limiting our ability to explore potential variations within the study population. Furthermore, the utilization of GWAS data in this study was limited to individuals of European descent, which restrict generalizability of the findings to other populations with different genetic backgrounds and environmental exposures. Dementia research remains largely void of diversity (Shaw et al., 2022). A systematic review of 96 RCTs to improve cognition related to dementia (total of 37,278 participants) found only 39 trials (39.4%) reported ethnicity and only 11.4% (95% CI, 7.5%–15.9%) of all participants were non‐White. Furthermore, there is also a lack of ethno‐racial reporting among participants in brain health prevention RCT trials. Specific recruitment strategies can help increase the inclusion of these populations (Perales‐Puchalt et al., 2020).

In conclusion, the present MR study demonstrated a significant correlation between genetically determined higher level of added salt in food and a higher risk of dementia. The association also significant in cognitive performance, dementia in AD, and undefined dementia. However, this association was not significant for other specific types of dementia, such as VaD, FTD, and DLB. Adding salt in food also a possible association with AD. Our study contributes to the growing body of evidence on the impact of salt intake on cognitive health and highlights the need for further research to elucidate the mechanisms underlying these associations. Future studies should aim to address the limitations of our research, including larger sample sizes for special types of dementia, more precise measurement of salt intake like urine 24‐h sodium excretion, and diverse population representation, in order to provide a comprehensive understanding of the relationship between salt intake and dementia.

## AUTHOR CONTRIBUTIONS

**Ren Zhou**: Software; writing—original draft. **Fei Chen**: Writing—original draft. **Lei Zhang**: Visualization. **Yu Sun**: Project administration. **Rong Hu**: Resources. **Jia Yan**: Resources. **Hong Jiang**: Supervision.

## CONFLICT OF INTEREST STATEMENT

The authors declare no actual or potential conflicts of interest.

## FUNDING INFORMATION

This work was supported by the Science and Technology Commission of Shanghai Municipality (STCSM) (23YF1422700).

### PEER REVIEW

The peer review history for this article is available at https://publons.com/publon/10.1002/brb3.3516.

## DECLARATION OF GENERATIVE AI AND AI‐ASSISTED TECHNOLOGIES IN THE WRITING PROCESS

During the preparation of this work the authors used ChatGPT 3.5 in order to improve language and readability. After using this tool, the authors reviewed and edited the content as needed and take full responsibility for the content of the publication.

## Supporting information

Table S1 Information of different types of dementia.
Table S2 Summary data of SNP.
Table S3 Association between any dementia and adding salt in food with multivariable mendelian randomization.
Table S4 MR results and sensitivity analysis for association of adding salt in food and dementia risk after deleted the SNPs association with BMI.

Figure S1 Scatter plot depicts the results of mendelian randomization (MR) analyses investigating the association between dietary salt intake and dementia. Each line in the plot represents a different MR method, and the slope of each line represents the estimated association between the two variables: (A) scatter plot between added salt in food and vascular dementia; (B) scatter plot between added salt in food and frontotemporal dementia; (C) scatter plot between added salt in food and dementia with Lewy bodies; (D) scatter plot between added salt in food and Parkinson's disease.

Figure S2 The figure displays the results of a leave‐one‐out analysis in mendelian randomization (MR). Each black line in the figure corresponds to the outcome of the MR analysis when one single nucleotide polymorphism (SNP) is removed from the analysis, whereas the remaining SNPs are used on the left: (A) leave‐one‐out analysis between added salt in food and vascular dementia; (B) leave‐one‐out analysis between added salt in food and frontotemporal dementia; (C) leave‐one‐out analysis between added salt in food and dementia with Lewy bodies; (D) leave‐one‐out analysis between added salt in food and Parkinson's disease.

Figure S3 Funnel plot shows the estimates of precision (1/SE) and Wald ratios for each SNP: (A) funnel plot on the effect of added salt in food and any dementia; (B) funnel plot on the effect of added salt in food and cognitive performance; (C) funnel plot on the effect of added salt in food and Alzheimer's disease; (D) funnel plot on the effect of added salt in food and dementia in Alzheimer's disease; (E) funnel plot on the effect of added salt in food and undefined dementia; (F) funnel plot on the effect of added salt in food and vascular dementia; (G) funnel plot on the effect of added salt in food and frontotemporal dementia; (H) funnel plot on the effect of added salt in food and dementia with Lewy bodies; (I) funnel plot on the effect of added salt in food and Parkinson's disease.

## Data Availability

The data supporting the findings of this study are available in IEU open GWAS project websites (https://gwas.mrcieu.ac.uk/) and can also obtained from the corresponding author upon reasonable request.
