# Supplementary material for: Association between adding salt in food and dementia in European descent: A mendelian randomization study
Source: Brain Behav. 2024 May 3;14(5):e3516. doi: 10.1002/brb3.3516 (PMC11069030; doi:10.1002/brb3.3516)
Supplement: Supplementary file 1 — Table S1 Information of different types of dementia. Table S2 Summary data of SNP. Table S3 Association between any dementia and adding salt in food with multivariable mendelian randomization. Table S4 MR results and sensitivity analysis for association of adding salt in food and dementia risk after deleted the SNPs association with BMI. [file BRB3-14-e3516-s002.docx]

**Association between Adding Salt in Food and Dementia in European Descent: A Mendelian Randomization Study**

Ren Zhou^#^, Fei Chen^#^, Lei Zhang, Yu Sun, Rong Hu, Jia Yan^*^, and Hong Jiang^*^

Department of Anesthesiology, The Ninth People’s Hospital of Shanghai, Jiao Tong University School of Medicine, Shanghai, 200011, PR China

*** Corresponding author**

Hong Jiang

The Ninth People’s Hospital of Shanghai, Jiao Tong University School of Medicine

No. 639 Zhizaoju Road, Shanghai, 200011, PR China.

Email: [Jianghongjiuyuan@163.com](mailto:Jianghongjiuyuan@163.com)

Rong Hu

The Ninth People’s Hospital of Shanghai, Jiao Tong University School of Medicine

No. 639 Zhizaoju Road, Shanghai, 200011, PR China.

Email: mzkyanj@163.com

#: Ren Zhou and Fei Chen contributed equally to this work.

| **Table S1: Information of different types of dementia** | | |
| --- | --- | --- |
| Types | Defined | Link of GWAS data information |
| Any Type Dementia | Dementia is a term for several diseases that affect memory, thinking, and the ability to perform daily activities. The illness gets worse over time. It mainly affects older people but not all people will get it as they age. | <https://r5.risteys.finngen.fi/phenocode/KRA_PSY_DEMENTIA> |
| Dementia with Lewy Bodies | Lewy body dementia is the second most common type of dementia after Alzheimer's disease. Protein deposits called Lewy bodies develop in nerve cells in the brain. The protein deposits affect brain regions involved in thinking, memory and movement. This condition is also known as dementia with Lewy bodies. | <https://pubmed.ncbi.nlm.nih.gov/33589841/> |
| Dementia in Alzheimer's Disease | Refer to Alzheimer's Disease section | <https://r5.risteys.finngen.fi/phenocode/F5_ALZHDEMENT> |
| Vascular Dementia | A degenerative vascular disorder affecting the brain. It is caused by the blockage of the blood supply to the brain. It is manifested with decline of memory and cognitive functions. | <https://r5.risteys.finngen.fi/phenocode/F5_VASCDEM> |
| Frontotemporal Dementia | Frontotemporal dementia (FTD) is an umbrella term for a group of brain diseases that mainly affect the frontal and temporal lobes of the brain. These areas of the brain are associated with personality, behavior and language. | <https://pubmed.ncbi.nlm.nih.gov/20154673/> |
| Undefined dementia | An acquired organic mental disorder with loss of intellectual abilities of sufficient severity to interfere with social or occupational functioning. The dysfunction is multifaceted and involves memory, behavior, personality, judgment, attention, spatial relations, language, abstract thought, and other executive functions. The intellectual decline is usually progressive, and initially spares the level of consciousness. | <https://r5.risteys.finngen.fi/phenocode/F5_Dementia_U> |
| Alzheimer's Disease | Alzheimer disease is a primary degenerative cerebral disease of unknown etiology with characteristic neuropathological and neurochemical features. The disorder is usually insidious in onset and develops slowly but steadily over a period of several years. | <https://r5.risteys.finngen.fi/phenocode/G6_ALZHEIMER> |
| Parkinson's disease | A progressive degenerative disorder of the central nervous system characterized by loss of dopamine producing neurons in the substantia nigra and the presence of Lewy bodies in the substantia nigra and locus coeruleus. Signs and symptoms include tremor which is most pronounced during rest, muscle rigidity, slowing of the voluntary movements, a tendency to fall back, and a mask-like facial expression. | <https://r5.risteys.finngen.fi/phenocode/G6_PARKINSON> |
| The define of different types of dementia were reference to Finn database website (https://r5.risteys.finngen.fi), Mayo Clinic Website (https://www.mayoclinic.org/) and WHO Website (https://www.who.int/). | | |

| **Table S2: Summary data of SNP** | | | | | | | | | | | |
| --- | --- | --- | --- | --- | --- | --- | --- | --- | --- | --- | --- |
| Included or not | SNP | pos | chr | pval | beta | se | effect allele | other allele | eaf | F value | Reason for excluded |
| Included | rs10128297 | 86845915 | 10 | 4.49997e-09 | -0.0105548 | 0.0018 | T | C | 0.402787 | 47.06333 | NA |
| Included | rs1045411 | 31033232 | 13 | 4.00037e-14 | -0.0150663 | 0.0019926 | T | C | 0.268305 | 60.53652 | NA |
| Included | rs10736951 | 281710 | 10 | 7.00003e-10 | -0.0113624 | 0.001843 | C | G | 0.635537 | 49.46147 | NA |
| Included | rs10752999 | 187279690 | 1 | 3.50026e-11 | 0.0127624 | 0.0019272 | C | A | 0.702035 | 54.13652 | NA |
| Included | rs10971930 | 34116083 | 9 | 4.49997e-09 | 0.0155012 | 0.0026436 | C | T | 0.127418 | 47.88237 | NA |
| Included | rs11022746 | 2442707 | 11 | 6.29999e-10 | 0.0118271 | 0.0019132 | G | T | 0.681941 | 50.50377 | NA |
| Included | rs11075194 | 13515170 | 16 | 4e-08 | -0.0098166 | 0.0017874 | G | A | 0.425797 | 44.10469 | NA |
| Included | rs11082431 | 42781374 | 18 | 3.79997e-08 | 0.0105645 | 0.0019207 | T | C | 0.301904 | 44.8918 | NA |
| Included | rs11210985 | 44808461 | 1 | 7.19946e-18 | -0.0156565 | 0.0018181 | A | G | 0.3791 | 68.84093 | NA |
| Included | rs11761254 | 1858651 | 7 | 3.80014e-12 | -0.0176809 | 0.0025459 | T | C | 0.136978 | 55.65221 | NA |
| Included | rs12094804 | 201733115 | 1 | 2e-08 | 0.0205059 | 0.0036558 | G | A | 0.061754 | 45.78654 | NA |
| Included | rs12501838 | 54972890 | 4 | 1.80011e-12 | 0.0146672 | 0.0020816 | T | A | 0.23366 | 57.63783 | NA |
| Included | rs12563932 | 163859836 | 1 | 4.49997e-08 | -0.0284929 | 0.00521 | C | A | 0.029478 | 43.92068 | NA |
| Included | rs12579997 | 110046112 | 12 | 8.60003e-12 | 0.0160311 | 0.0023479 | C | G | 0.170455 | 55.83563 | NA |
| Included | rs12658060 | 51375189 | 5 | 2.49977e-38 | -0.0274145 | 0.0021176 | C | T | 0.222971 | 102.8313 | NA |
| Included | rs12789951 | 99719509 | 11 | 7.00003e-11 | -0.0117174 | 0.0017969 | T | C | 0.411036 | 52.2869 | NA |
| Included | rs12988960 | 164378068 | 2 | 2.80027e-15 | 0.014981 | 0.0018965 | C | G | 0.315972 | 64.69931 | NA |
| Included | rs13084934 | 7455198 | 3 | 4.39997e-08 | -0.0096756 | 0.0017674 | T | A | 0.494691 | 43.96486 | NA |
| Included | rs13131880 | 178963783 | 4 | 2.99999e-09 | -0.0126193 | 0.0021275 | C | T | 0.220413 | 47.60287 | NA |
| Included | rs1728779 | 68567500 | 16 | 3.50002e-08 | 0.0098708 | 0.0017892 | G | A | 0.436092 | 45.02709 | NA |
| Included | rs1823011 | 85698520 | 5 | 3.89996e-08 | -0.0102255 | 0.0018612 | G | A | 0.659939 | 44.1214 | NA |
| Included | rs1895951 | 108268689 | 12 | 4.79999e-08 | -0.0116911 | 0.0021422 | C | T | 0.220794 | 43.83081 | NA |
| Included | rs2263636 | 63495365 | 2 | 1e-08 | 0.0112558 | 0.0019645 | C | A | 0.719001 | 46.77702 | NA |
| Included | rs2339234 | 170612546 | 5 | 1.59993e-20 | -0.0176416 | 0.0019001 | A | G | 0.683186 | 74.14752 | NA |
| Included | rs2457427 | 22746069 | 8 | 1.09999e-10 | -0.0165822 | 0.0025695 | C | T | 0.863856 | 51.75275 | NA |
| Included | rs2463710 | 72866597 | 6 | 4.90004e-08 | 0.0119523 | 0.0021912 | A | T | 0.753885 | 44.51568 | NA |
| Included | rs2506738 | 104003425 | 6 | 5.00035e-17 | -0.0151014 | 0.0018007 | G | A | 0.401141 | 67.06363 | NA |
| Included | rs2693687 | 99672917 | 14 | 1.7e-10 | 0.0116279 | 0.0018205 | T | C | 0.393736 | 52.19811 | NA |
| Included | rs2736748 | 60815608 | 3 | 1.80011e-38 | 0.0280118 | 0.0021596 | G | A | 0.789193 | 107.0326 | NA |
| Included | rs2835623 | 38511531 | 21 | 4.39997e-08 | 0.0226771 | 0.0041436 | T | C | 0.04766 | 44.66421 | NA |
| Included | rs28366169 | 32238496 | 6 | 2.59998e-10 | -0.0208479 | 0.0032994 | A | G | 0.077319 | 50.68144 | NA |
| Included | rs2852348 | 37189171 | 18 | 1.59993e-13 | -0.0131167 | 0.0017774 | G | A | 0.458696 | 59.09983 | NA |
| Included | rs2899345 | 41815609 | 22 | 2.5e-08 | 0.0098867 | 0.001773 | C | T | 0.504298 | 45.51558 | NA |
| Included | rs324018 | 57487729 | 12 | 3.29997e-08 | 0.0100101 | 0.0018116 | G | T | 0.613528 | 45.09959 | NA |
| Included | rs329670 | 133794599 | 11 | 6.29999e-09 | -0.0152915 | 0.0026328 | C | T | 0.870289 | 46.62198 | NA |
| Included | rs33137 | 158064276 | 5 | 9.3994e-12 | -0.0120839 | 0.0017732 | T | C | 0.493871 | 54.62218 | NA |
| Included | rs34906832 | 101738082 | 4 | 2.39999e-09 | 0.0150976 | 0.0025301 | G | A | 0.140896 | 48.73537 | NA |
| Included | rs35099536 | 9516653 | 6 | 5.79963e-16 | 0.0259587 | 0.0032075 | C | A | 0.08461 | 66.30462 | NA |
| Included | rs35142265 | 38269759 | 17 | 2.09991e-12 | 0.0150134 | 0.0021364 | G | A | 0.218202 | 57.48419 | NA |
| Included | rs35271178 | 17411020 | 11 | 1.09999e-08 | 0.010244 | 0.0017909 | T | C | 0.588507 | 46.69839 | NA |
| Included | rs35702851 | 841284 | 11 | 2.59998e-08 | 0.0121964 | 0.0021906 | C | G | 0.204203 | 45.44481 | NA |
| Included | rs3890316 | 94242898 | 4 | 2.80001e-08 | -0.0113326 | 0.0020416 | A | G | 0.249954 | 44.57229 | NA |
| Included | rs400750 | 18634962 | 3 | 3.29989e-20 | 0.016712 | 0.0018147 | G | T | 0.616712 | 75.57352 | NA |
| Included | rs4236065 | 12167286 | 6 | 3.79997e-08 | -0.0111709 | 0.0020305 | C | T | 0.255885 | 44.18055 | NA |
| Included | rs4595499 | 62792589 | 10 | 1.20005e-21 | -0.0172775 | 0.0018077 | T | C | 0.391888 | 76.29633 | NA |
| Included | rs4912891 | 142402117 | 5 | 2.99999e-08 | 0.0106552 | 0.001923 | C | T | 0.691882 | 45.22552 | NA |
| Included | rs4948275 | 63136622 | 10 | 7.39946e-13 | 0.0127146 | 0.0017727 | T | C | 0.481851 | 58.68379 | NA |
| Included | rs4981196 | 33985724 | 14 | 3.29997e-10 | -0.0114561 | 0.0018225 | C | A | 0.635898 | 50.42189 | NA |
| Included | rs526210 | 90131593 | 4 | 2.80001e-08 | 0.010095 | 0.0018187 | A | G | 0.542868 | 45.30496 | NA |
| Included | rs55897719 | 212590841 | 2 | 1.10002e-12 | 0.0135021 | 0.001896 | A | C | 0.316817 | 58.25976 | NA |
| Included | rs586716 | 22488678 | 9 | 1.10002e-15 | 0.0143673 | 0.0017934 | A | G | 0.414681 | 65.62613 | NA |
| Included | rs62098445 | 58819108 | 18 | 1e-16 | -0.0158642 | 0.0019115 | A | C | 0.318657 | 66.37519 | NA |
| Included | rs6416794 | 51181735 | 16 | 4.90004e-08 | -0.0119996 | 0.0022003 | C | T | 0.798724 | 43.79853 | NA |
| Included | rs6443950 | 183817727 | 3 | 8.49963e-15 | -0.0142051 | 0.0018306 | T | A | 0.631504 | 62.10839 | NA |
| Included | rs667128 | 10961174 | 12 | 5.99998e-09 | 0.0154255 | 0.0026523 | T | C | 0.126584 | 47.4889 | NA |
| Included | rs6695915 | 44010571 | 1 | 2.90001e-08 | -0.0166292 | 0.0029984 | G | A | 0.095551 | 44.53552 | NA |
| Included | rs6776248 | 33420650 | 3 | 1.40001e-08 | 0.0113408 | 0.0019979 | C | T | 0.26336 | 46.33875 | NA |
| Included | rs6804929 | 119833578 | 3 | 7.39997e-09 | 0.0167058 | 0.0028892 | A | G | 0.105016 | 47.21069 | NA |
| Included | rs6987313 | 71570989 | 8 | 2e-08 | 0.009907 | 0.0017658 | C | T | 0.520156 | 45.79766 | NA |
| Included | rs7021360 | 11592801 | 9 | 2.30001e-08 | -0.0101193 | 0.0018101 | A | C | 0.388225 | 44.88822 | NA |
| Included | rs7110845 | 120175749 | 11 | 1.09999e-10 | -0.0115376 | 0.0017863 | G | A | 0.579174 | 51.79681 | NA |
| Included | rs72807804 | 82861212 | 16 | 1.7e-08 | 0.0120641 | 0.002139 | T | C | 0.218782 | 46.04115 | NA |
| Included | rs73040343 | 18466059 | 3 | 1.2e-08 | -0.0103509 | 0.0018142 | G | A | 0.381434 | 45.80581 | NA |
| Included | rs736935 | 60377674 | 3 | 6.79986e-17 | 0.0156881 | 0.0018787 | T | C | 0.336413 | 68.43749 | NA |
| Included | rs7465705 | 23190841 | 9 | 6.79986e-11 | -0.0118468 | 0.0018158 | A | G | 0.384538 | 52.31555 | NA |
| Included | rs7581335 | 143958864 | 2 | 3.79997e-09 | 0.014931 | 0.0025332 | T | A | 0.140518 | 48.13323 | NA |
| Included | rs7591518 | 145632077 | 2 | 5.10035e-27 | -0.0207723 | 0.0019298 | C | T | 0.298453 | 85.77542 | NA |
| Included | rs7670308 | 67230580 | 4 | 1e-10 | -0.0116397 | 0.0018012 | A | G | 0.409195 | 51.82151 | NA |
| Included | rs7673170 | 18251259 | 4 | 2.70023e-12 | -0.0125967 | 0.0018013 | A | G | 0.395273 | 56.03472 | NA |
| Included | rs7927679 | 126757949 | 11 | 2.29985e-17 | 0.0149539 | 0.001764 | T | C | 0.504024 | 69.4916 | NA |
| Included | rs7982263 | 80061074 | 13 | 2.90001e-09 | 0.0106084 | 0.0017875 | C | T | 0.581981 | 48.46765 | NA |
| Included | rs8022455 | 98883534 | 14 | 3.40001e-10 | -0.0111417 | 0.001774 | C | T | 0.54702 | 50.37892 | NA |
| Included | rs8040685 | 54936197 | 15 | 2.30001e-08 | -0.0154071 | 0.0027581 | T | C | 0.884465 | 44.85353 | NA |
| Included | rs8097544 | 1839564 | 18 | 2.39999e-08 | 0.0140302 | 0.0025128 | G | A | 0.145366 | 45.57551 | NA |
| Included | rs868720 | 116566836 | 5 | 9.60064e-17 | 0.0159938 | 0.0019247 | C | G | 0.305451 | 68.10166 | NA |
| Included | rs9278020 | 33326699 | 6 | 4.79954e-16 | 0.0232222 | 0.0028615 | A | G | 0.10619 | 66.4888 | NA |
| Included | rs9317406 | 64819020 | 13 | 4.39997e-09 | -0.0107015 | 0.0018238 | T | C | 0.376115 | 47.0945 | NA |
| Included | rs9375448 | 126896782 | 6 | 3.40017e-11 | -0.0116787 | 0.0017616 | T | A | 0.492981 | 53.15059 | NA |
| Included | rs9569747 | 58370948 | 13 | 8.60003e-11 | -0.0125594 | 0.0019355 | G | T | 0.298073 | 52.03395 | NA |
| Included | rs961044 | 87608094 | 14 | 2.99999e-08 | -0.0140077 | 0.0025273 | T | C | 0.857621 | 44.50768 | NA |
| Included | rs9611875 | 43153807 | 22 | 1.29987e-48 | 0.0662007 | 0.0045187 | G | A | 0.039616 | 121.1891 | NA |
| Included | rs9667150 | 31010455 | 11 | 1.50003e-12 | 0.012589 | 0.0017806 | A | G | 0.559523 | 57.83595 | NA |
| Included | rs976179 | 198880378 | 2 | 1.2e-08 | 0.0100673 | 0.0017649 | T | A | 0.485324 | 46.5684 | NA |
| Included | rs99780 | 61596633 | 11 | 3.59998e-09 | 0.0109071 | 0.0018476 | T | C | 0.350239 | 48.20993 | NA |
| Excluded | rs11126666 | 26928811 | 2 | 2.90001e-09 | -0.0119879 | 0.0020191 | A | G | 0.255562 |  | Diastolic blood pressure |
| Excluded | rs13400612 | 138318976 | 2 | 2.99985e-23 | 0.0202846 | 0.0020425 | G | C | 0.246906 |  | Diastolic blood pressure |
| Excluded | rs11130206 | 49596593 | 3 | 1.20005e-11 | -0.0120324 | 0.0017755 | G | C | 0.432321 |  | Diastolic blood pressure |
| Excluded | rs2521501 | 91437388 | 15 | 2e-09 | -0.0113954 | 0.0019004 | T | A | 0.322086 |  | Diastolic blood pressure |
| Excluded | rs2547040 | 24736408 | 16 | 1.2e-08 | -0.0127081 | 0.0022294 | C | G | 0.805074 |  | Diastolic blood pressure |
| Excluded | rs429358 | 45411941 | 19 | 2.90001e-10 | -0.0153977 | 0.002442 | C | T | 0.154168 |  | Diastolic blood pressure |
| Excluded | rs17805497 | 177070172 | 2 | 1.50003e-11 | 0.0124815 | 0.0018502 | C | T | 0.345071 |  | Systolic blood pressure |
| Excluded | rs10883796 | 104655315 | 10 | 3.29997e-09 | 0.0114406 | 0.0019342 | A | G | 0.294358 |  | Systolic blood pressure |
| Excluded | rs491907 | 50908697 | 15 | 4.60002e-10 | 0.0109863 | 0.001763 | G | A | 0.511555 |  | Systolic blood pressure |
| Excluded | rs528301 | 45154908 | 2 | 6.59933e-18 | 0.0152609 | 0.0017701 | A | G | 0.554846 |  | Alcohol intake frequency |
| Excluded | rs6780346 | 85593584 | 3 | 1.9002e-20 | -0.0168372 | 0.0018172 | T | C | 0.620997 |  | Alcohol intake frequency |
| Excluded | rs1726866 | 141672705 | 7 | 7.70016e-51 | 0.0265437 | 0.00177 | A | G | 0.552077 |  | Alcohol intake frequency |
| Excluded | rs4739105 | 64496159 | 8 | 1.29987e-12 | 0.0154648 | 0.0021801 | C | T | 0.787781 |  | Alcohol intake frequency |
| Excluded | rs264932 | 104288396 | 2 | 7.29995e-09 | -0.0104199 | 0.0018018 | G | A | 0.605889 |  | Ever smoked |
| Excluded | rs9843358 | 83242378 | 3 | 6.20012e-16 | 0.0189528 | 0.0023442 | T | C | 0.171903 |  | Ever smoked |
| Excluded | rs9835772 | 85766025 | 3 | 2.39999e-08 | 0.0114509 | 0.0020521 | T | A | 0.243664 |  | Ever smoked |
| Excluded | rs4235642 | 103818412 | 5 | 1.29987e-11 | 0.0122937 | 0.0018165 | G | A | 0.379691 |  | Ever smoked |
| Excluded | rs6887291 | 106438742 | 5 | 5.10035e-12 | 0.0126757 | 0.0018365 | G | T | 0.636126 |  | Ever smoked |
| Excluded | rs1008078 | 91189731 | 1 | 1.09999e-10 | 0.0116597 | 0.0018065 | T | C | 0.395512 |  | Educational attainment college completion |
| Excluded | rs4860797 | 67801134 | 4 | 1.09999e-10 | 0.011667 | 0.0018076 | A | G | 0.601276 |  | Years of educational attainment |
| Excluded | rs10140751 | 26985560 | 14 | 3.59998e-08 | -0.0112745 | 0.0020467 | T | G | 0.245951 |  | Age completed full time education |
| Excluded | rs4799949 | 35155910 | 18 | 4.49997e-09 | -0.0109799 | 0.0018721 | T | C | 0.667226 |  | Age completed full time education |
|  |  |  |  |  |  |  |  |  |  |  |  |

| **Table S3. Association between any dementia and adding salt in food with multivariable mendelian randomization** | | | | |
| --- | --- | --- | --- | --- |
| Outcome | Number of SNP | OR or β (95% CI) | P | FDR |
| Any Type Dementia | 73 | 1.56 (1.11, 2.19) | 0.010 | 0.034 |
| Cognitive performance | 76 | -0.141 (-0.253, -0.03) | 0.013 | 0.034 |
| Dementia with Lewy Bodies | 67 | 0.95 (0.47, 1.92) | 0.885 | 0.988 |
| Dementia in Alzheimer's Disease | 73 | 2.19 (1.25, 3.82) | 0.006 | 0.034 |
| Vascular Dementia | 73 | 1.60 (0.66, 3.87) | 0.301 | 0.452 |
| Frontotemporal Dementia | 31 | 0.50 (0.06, 3.89) | 0.509 | 0.654 |
| Undefined Dementia | 73 | 2.35 (1.18, 4.69) | 0.015 | 0.034 |
| Alzheimer's Disease | 73 | 1.44 (0.92, 2.25) | 0.107 | 0.192 |
| Parkinson's disease | 73 | 1.00 (0.57, 1.73) | 0.988 | 0.988 |
| GWAS data of smoking status(ukb-b-20261), drinking status (ukb-b-5779), education level (ukb-b-6134), and Energy (ukb-b-7323) were included in MVMR analysis. | | | | |

| **Table S4: MR results and sensitivity analysis for association of adding salt in food and dementia risk after deleted the SNPs association with BMI.** | | | | |
| --- | --- | --- | --- | --- |
| Outcome | Number of SNP | OR or β (95% CI) | P | FDR |
| Any Type Dementia | 68 | 1.83 (1.22, 2.74) | 0.003 | 0.022 |
| Cognitive performance | 70 | -0.099 (-0.196, -0.002) | 0.045 | 0.068 |
| Dementia with Lewy Bodies | 66 | 1.25 (0.61, 2.57) | 0.542 | 0.605 |
| Dementia in Alzheimer's Disease | 68 | 2.08 (1.09, 3.97) | 0.027 | 0.049 |
| Vascular Dementia | 68 | 3.23 (1.16, 9.04) | 0.025 | 0.049 |
| Frontotemporal Dementia | 31 | 0.54 (0.07, 4.04) | 0.550 | 0.605 |
| Undefined Dementia | 68 | 3.07 (1.41, 6.71) | 0.005 | 0.022 |
| Alzheimer's Disease | 68 | 1.83 (1.09, 3.08) | 0.023 | 0.049 |
| Parkinson's disease | 68 | 1.18 (0.62, 2.25) | 0.605 | 0.605 |
|  |  |  |  |  |


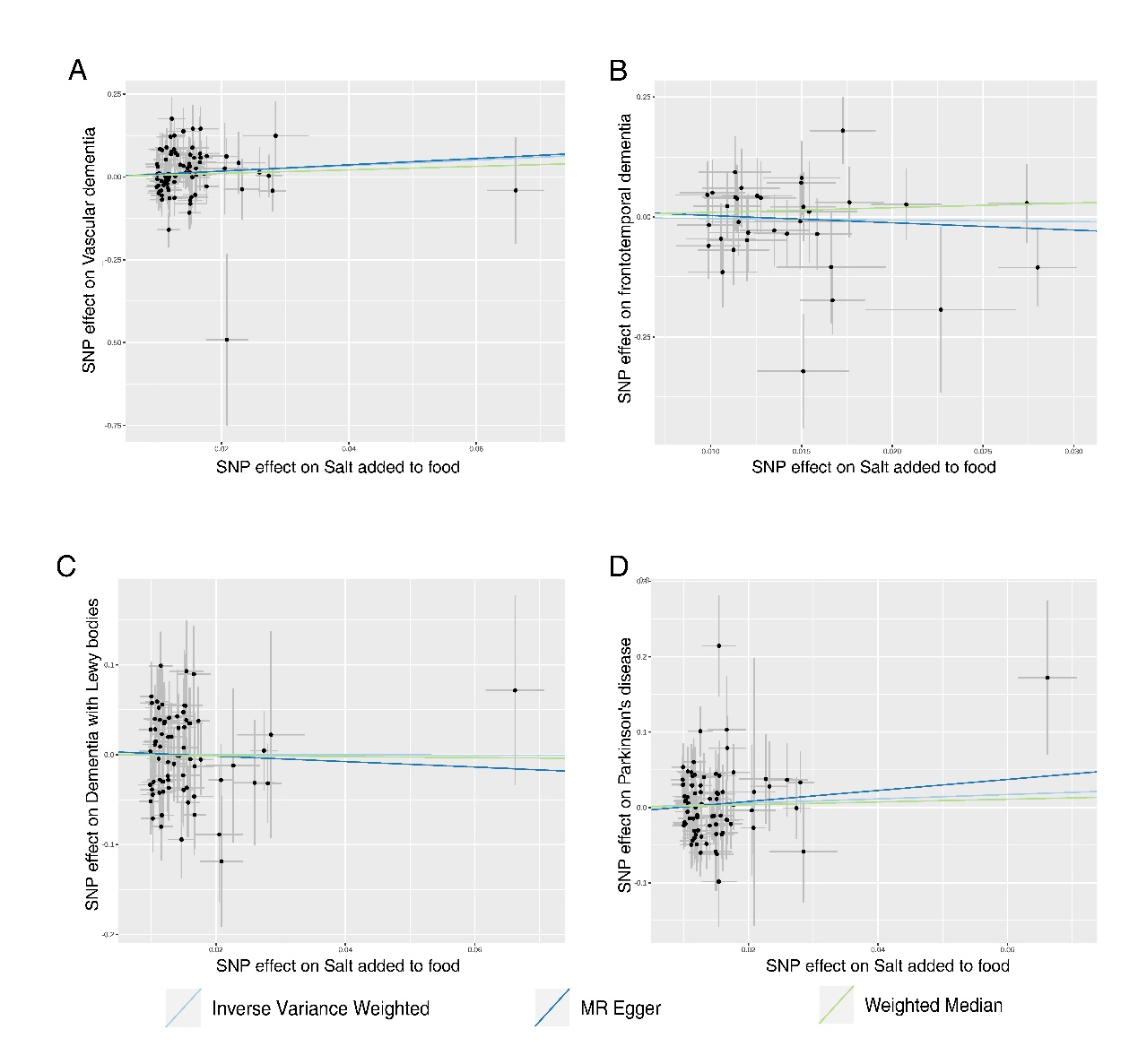
**Figure S1**


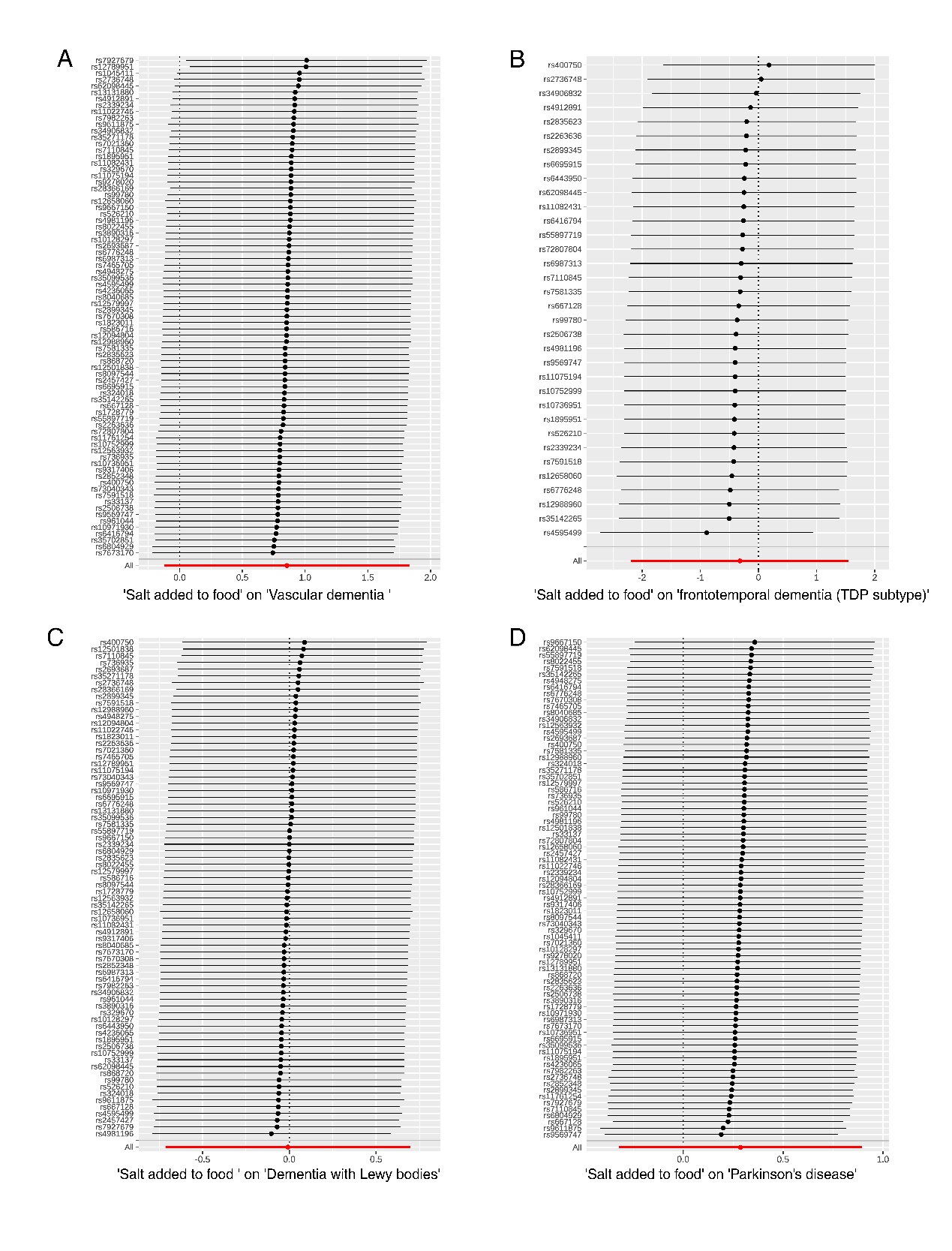
**Figure S2**

**Figure S3:**


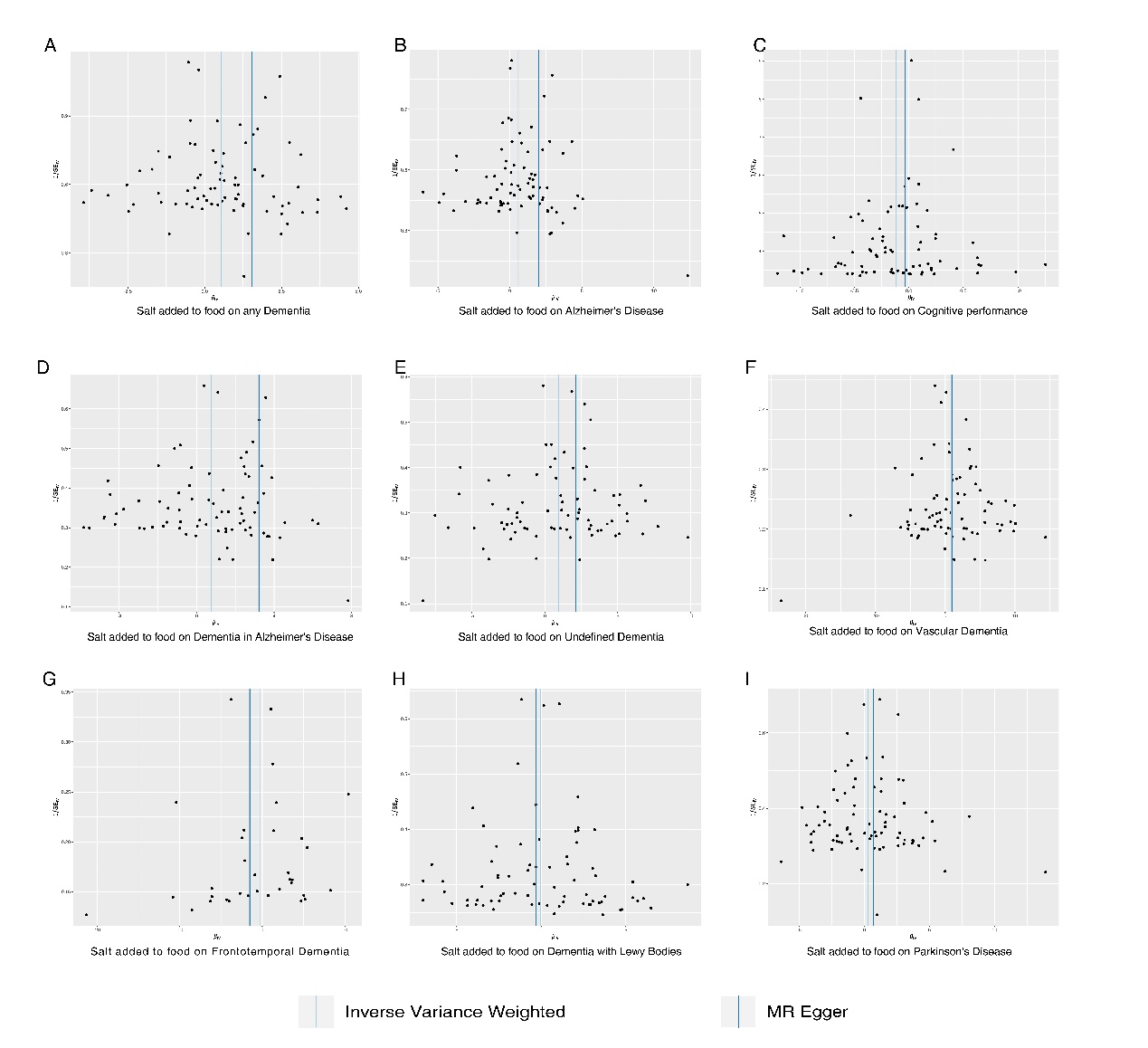


**Figure legends**

**Figure S1:** Scatter plot depicts the results of Mendelian randomization (MR) analyses investigating the association between dietary salt intake and dementia. Each line in the plot represents a different MR method, and the slope of each line represents the estimated association between the two variables. A: Scatter plot between added salt in food and vascular dementia; B: Scatter plot between added salt in food and frontotemporal dementia; C: Scatter plot between added salt in food and dementia with Lewy bodies; D: Scatter plot between added salt in food and Parkinson's disease.

**Figure S2:** The figure displays the results of a leave-one-out analysis in Mendelian randomization (MR). Each black line in the figure corresponds to the outcome of the MR analysis when one single nucleotide polymorphism (SNP) is removed from the analysis, while the remaining SNPs are used on the left. A: Leave-one-out analysis between added salt in food and vascular dementia; B: Leave-one-out analysis between added salt in food and frontotemporal dementia; C: Leave-one-out analysis between added salt in food and dementia with Lewy bodies; D: Leave-one-out analysis between added salt in food and Parkinson's disease.

**Figure S3:** Funnel plot shows the estimates of precision (1/SE) and Wald ratios for each SNP. A: Funnel plot on the effect of added salt in food and any dementia; B: Funnel plot on the effect of added salt in food and cognitive performance; C: Funnel plot on the effect of added salt in food and Alzheimer's disease; D: Funnel plot on the effect of added salt in food and dementia in Alzheimer's disease; E: Funnel plot on the effect of added salt in food and undefined dementia; F: Funnel plot on the effect of added salt in food and vascular dementia; G: Funnel plot on the effect of added salt in food and frontotemporal dementia; H: Funnel plot on the effect of added salt in food and dementia with Lewy bodies; I: Funnel plot on the effect of added salt in food and Parkinson's disease.
