# Supplementary figures and images for: Association between adding salt in food and dementia in European descent: A mendelian randomization study
Source: Brain Behav. 2024 May 3;14(5):e3516. doi: 10.1002/brb3.3516 (PMC11069030; doi:10.1002/brb3.3516)

A

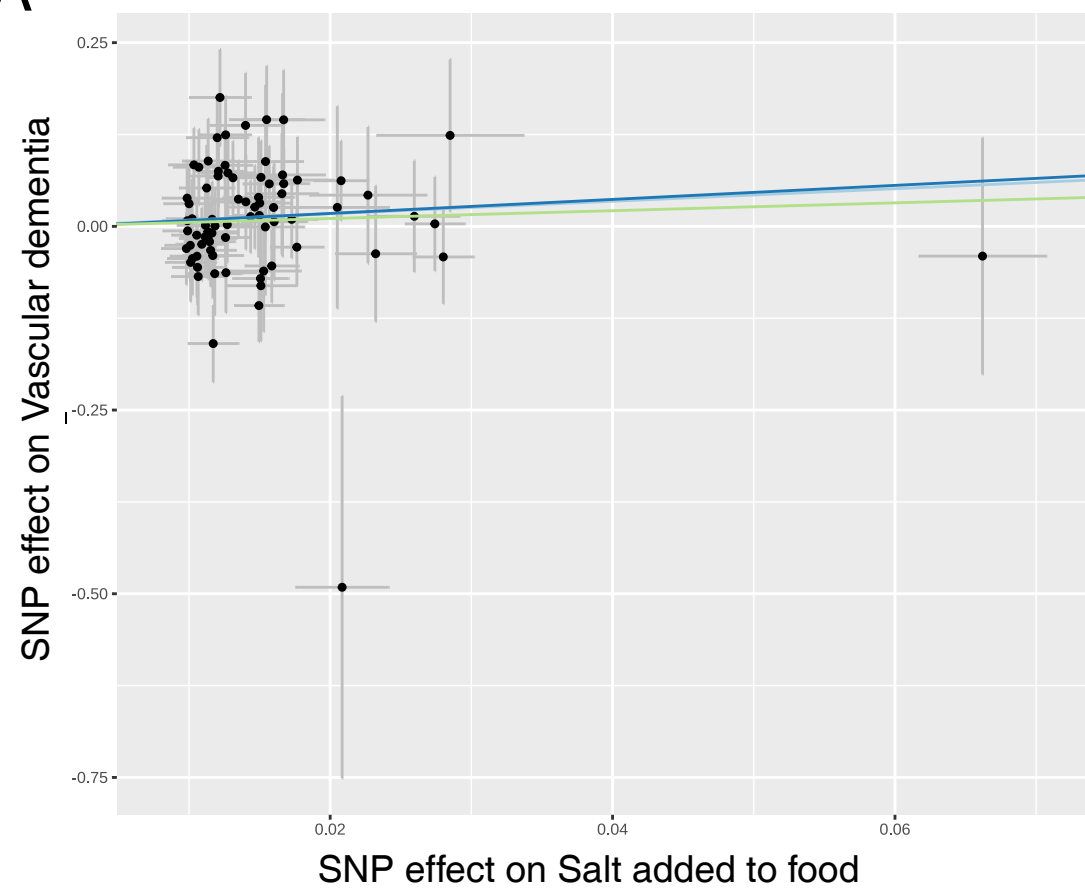

B

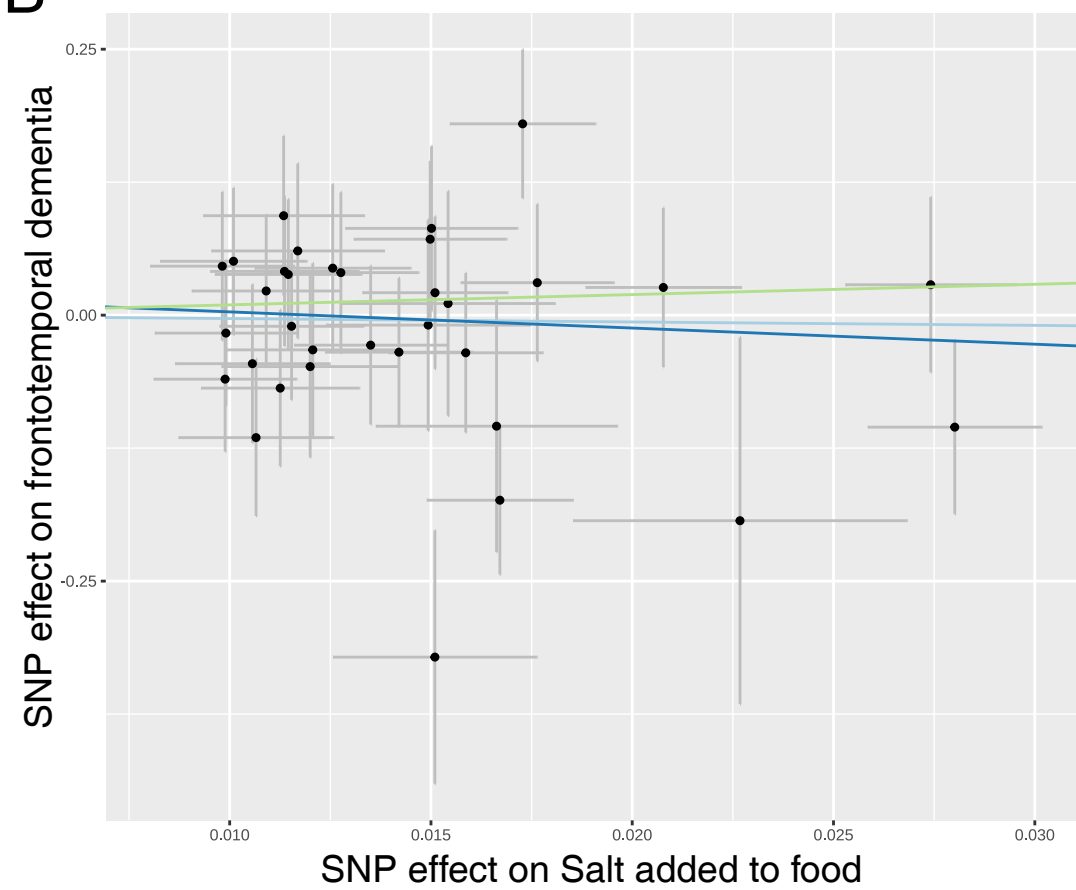

C

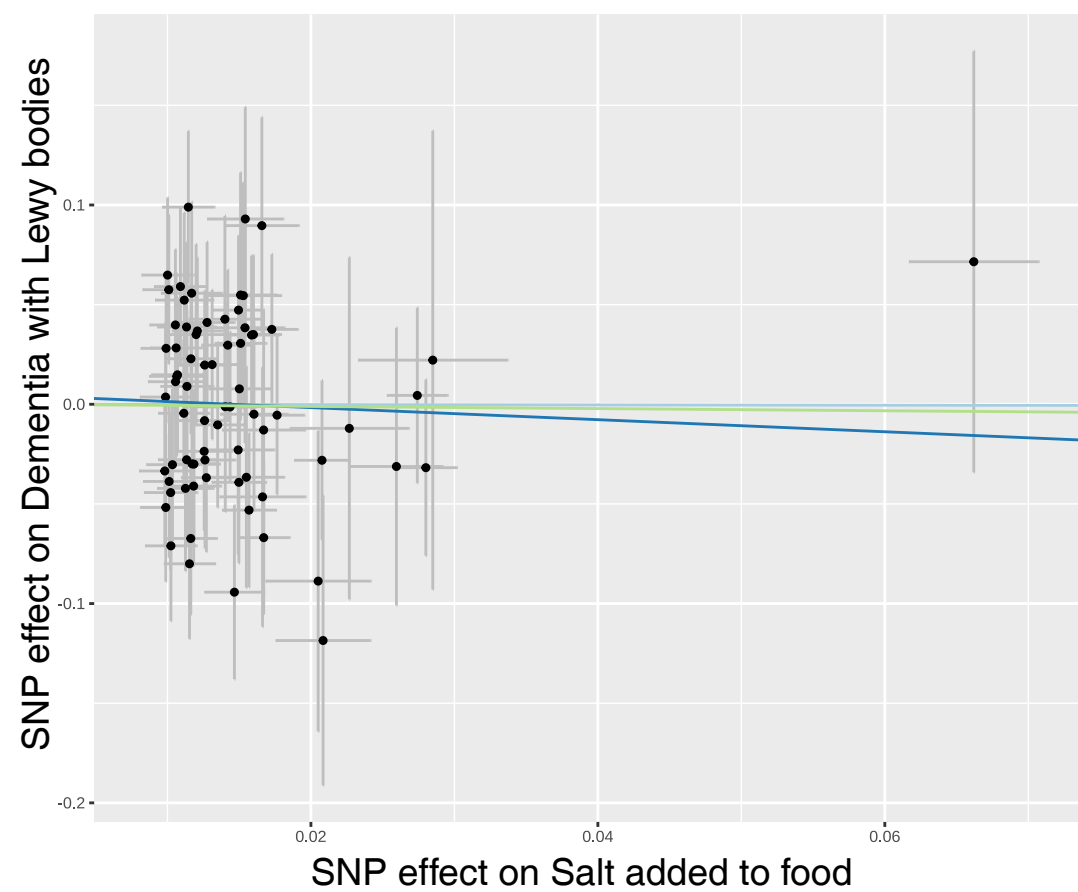

D

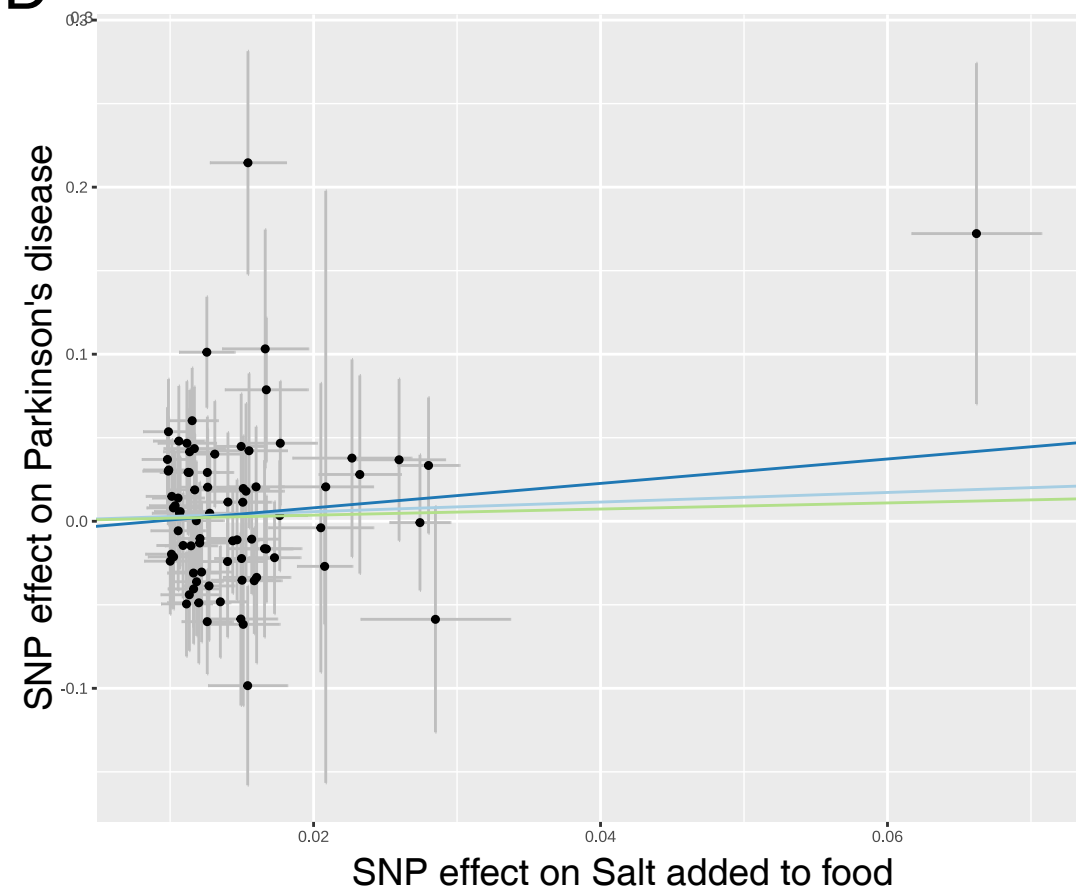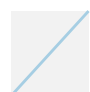

Inverse Variance Weighted

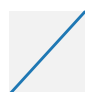

MR Egger

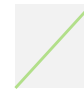

Weighted Median

Supplement: Supplementary file 2 — Figure S1 Scatter plot depicts the results of mendelian randomization (MR) analyses investigating the association between dietary salt intake and dementia. Each line in the plot represents a different MR method, and the slope of each line represents the estimated association between the two variables: (A) scatter plot between added salt in food and vascular dementia; (B) scatter plot between added salt in food and frontotemporal dementia; (C) scatter plot between added salt in food and dementia with Lewy bodies; (D) scatter plot between added salt in food and Parkinson's disease. [file BRB3-14-e3516-s003.pdf]

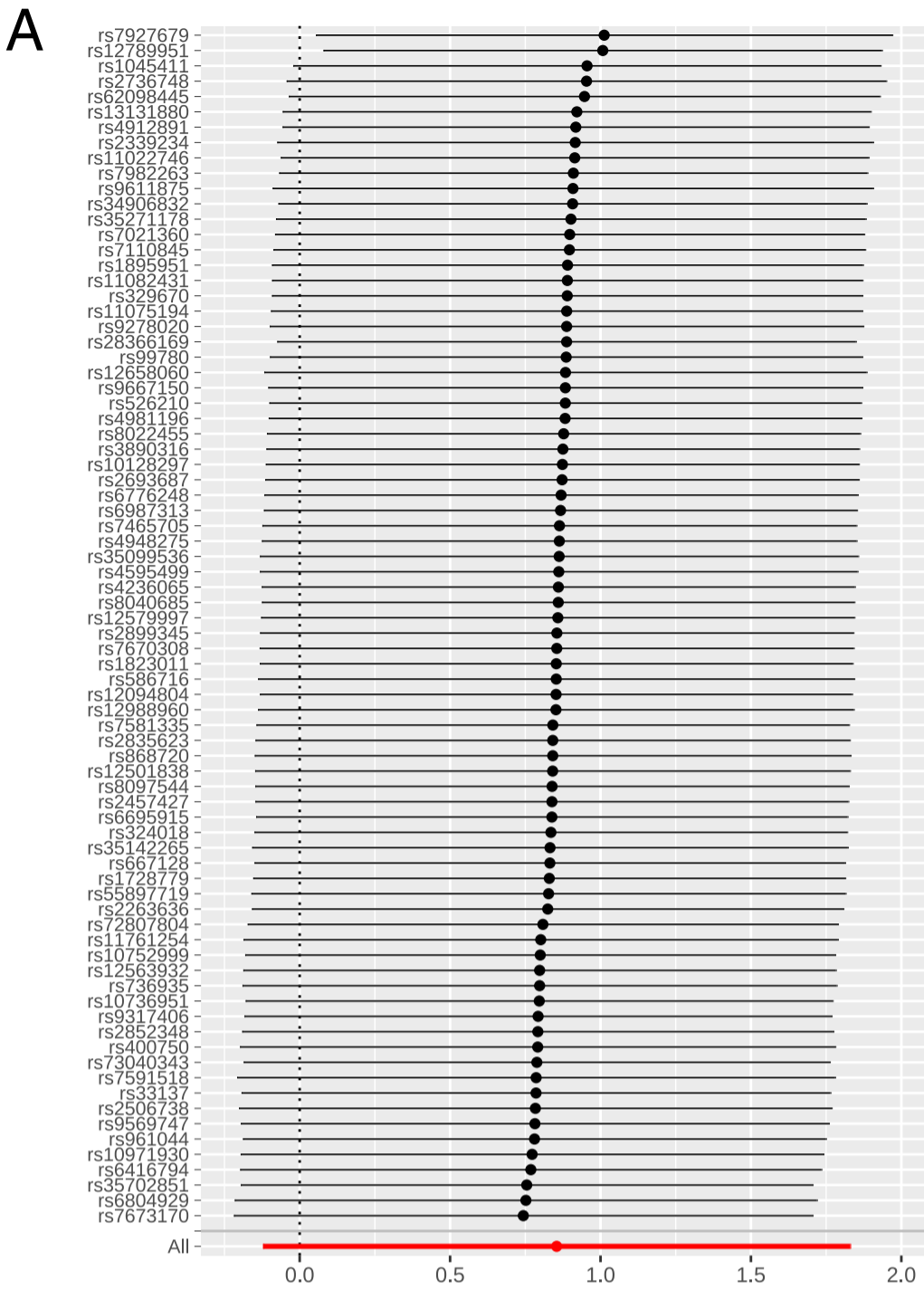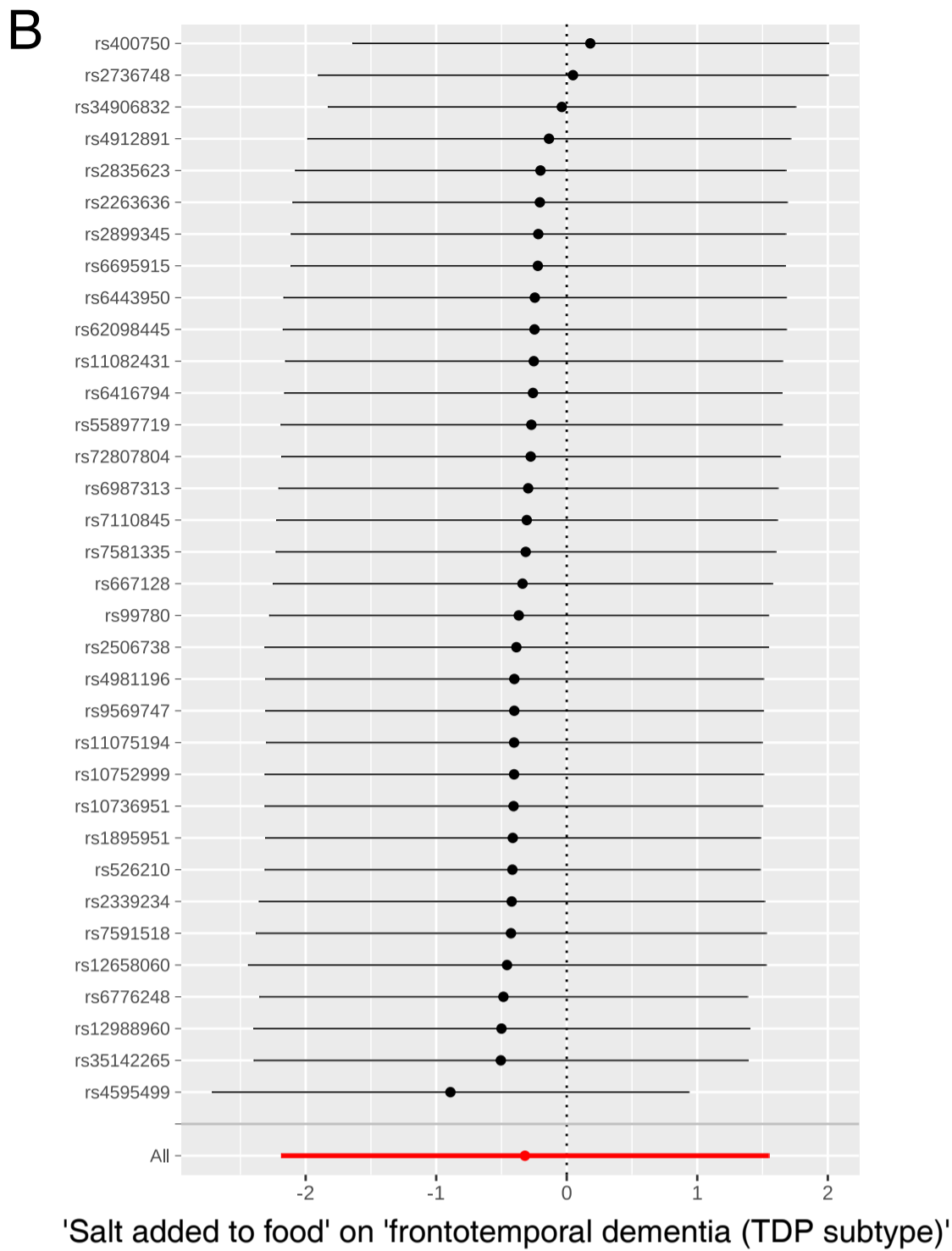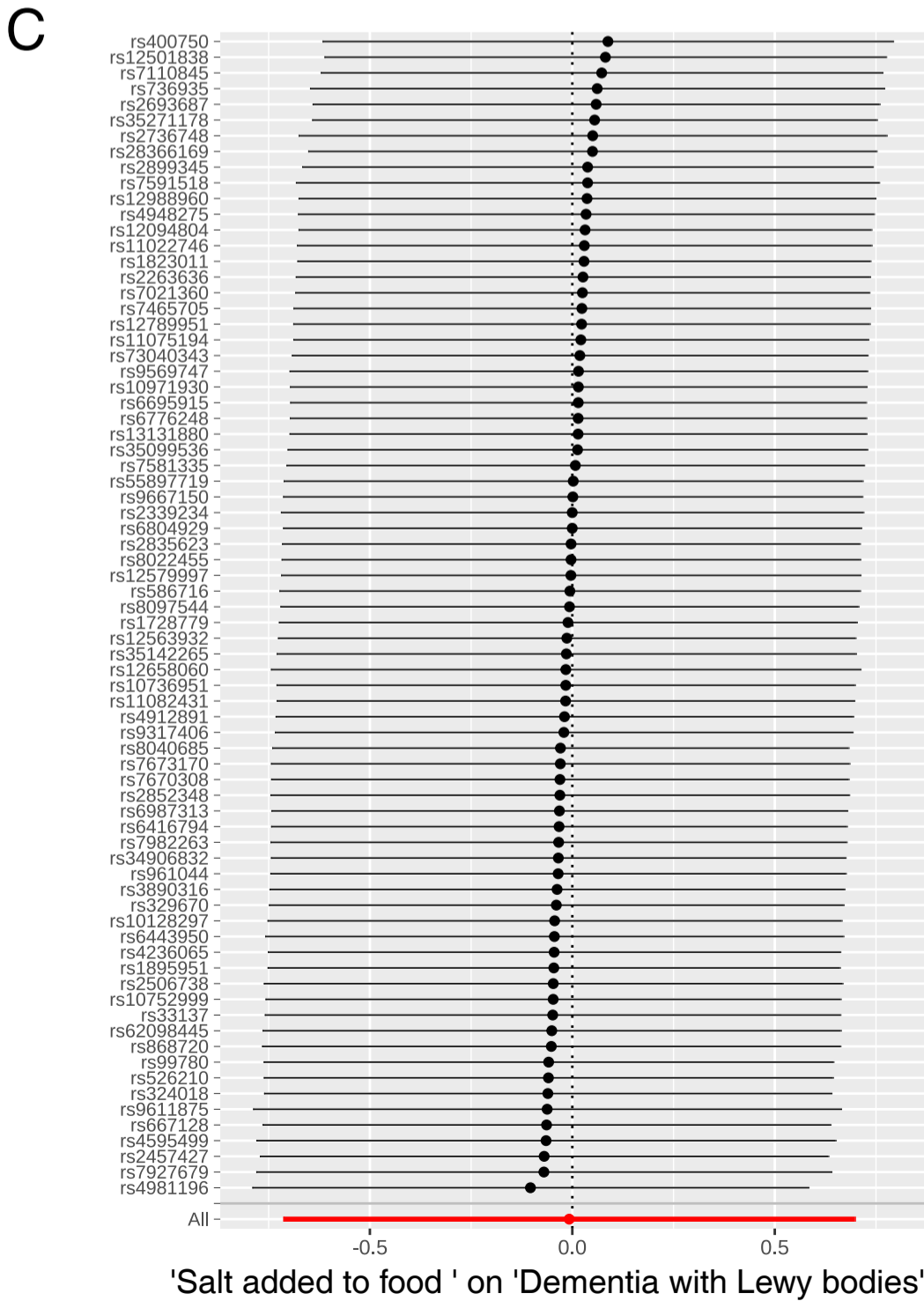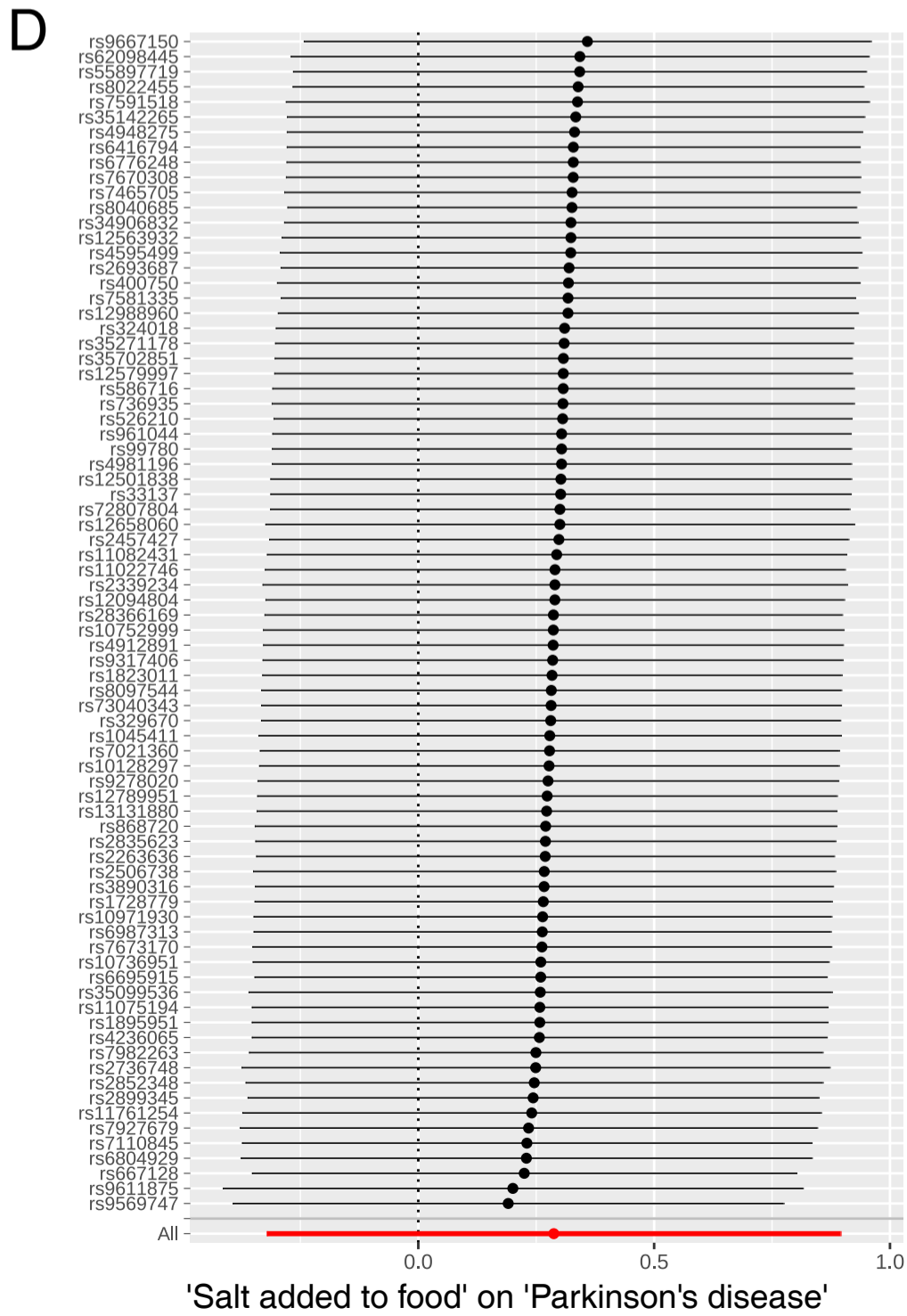

Supplement: Supplementary file 3 — Figure S2 The figure displays the results of a leave‐one‐out analysis in mendelian randomization (MR). Each black line in the figure corresponds to the outcome of the MR analysis when one single nucleotide polymorphism (SNP) is removed from the analysis, whereas the remaining SNPs are used on the left: (A) leave‐one‐out analysis between added salt in food and vascular dementia; (B) leave‐one‐out analysis between added salt in food and frontotemporal dementia; (C) leave‐one‐out analysis between added salt in food and dementia with Lewy bodies; (D) leave‐one‐out analysis between added salt in food and Parkinson's disease. [file BRB3-14-e3516-s004.pdf]
