# Supplementary material for: Association between adding salt in food and dementia in European descent: A mendelian randomization study
Source: Brain Behav. 2024 May 3;14(5):e3516. doi: 10.1002/brb3.3516 (PMC11069030; doi:10.1002/brb3.3516)
Supplement: Supplementary file 4 — Figure S3 Funnel plot shows the estimates of precision (1/SE) and Wald ratios for each SNP: (A) funnel plot on the effect of added salt in food and any dementia; (B) funnel plot on the effect of added salt in food and cognitive performance; (C) funnel plot on the effect of added salt in food and Alzheimer's disease; (D) funnel plot on the effect of added salt in food and dementia in Alzheimer's disease; (E) funnel plot on the effect of added salt in food and undefined dementia; (F) funnel plot on the effect of added salt in food and vascular dementia; (G) funnel plot on the effect of added salt in food and frontotemporal dementia; (H) funnel plot on the effect of added salt in food and dementia with Lewy bodies; (I) funnel plot on the effect of added salt in food and Parkinson's disease. [file BRB3-14-e3516-s001.pdf]

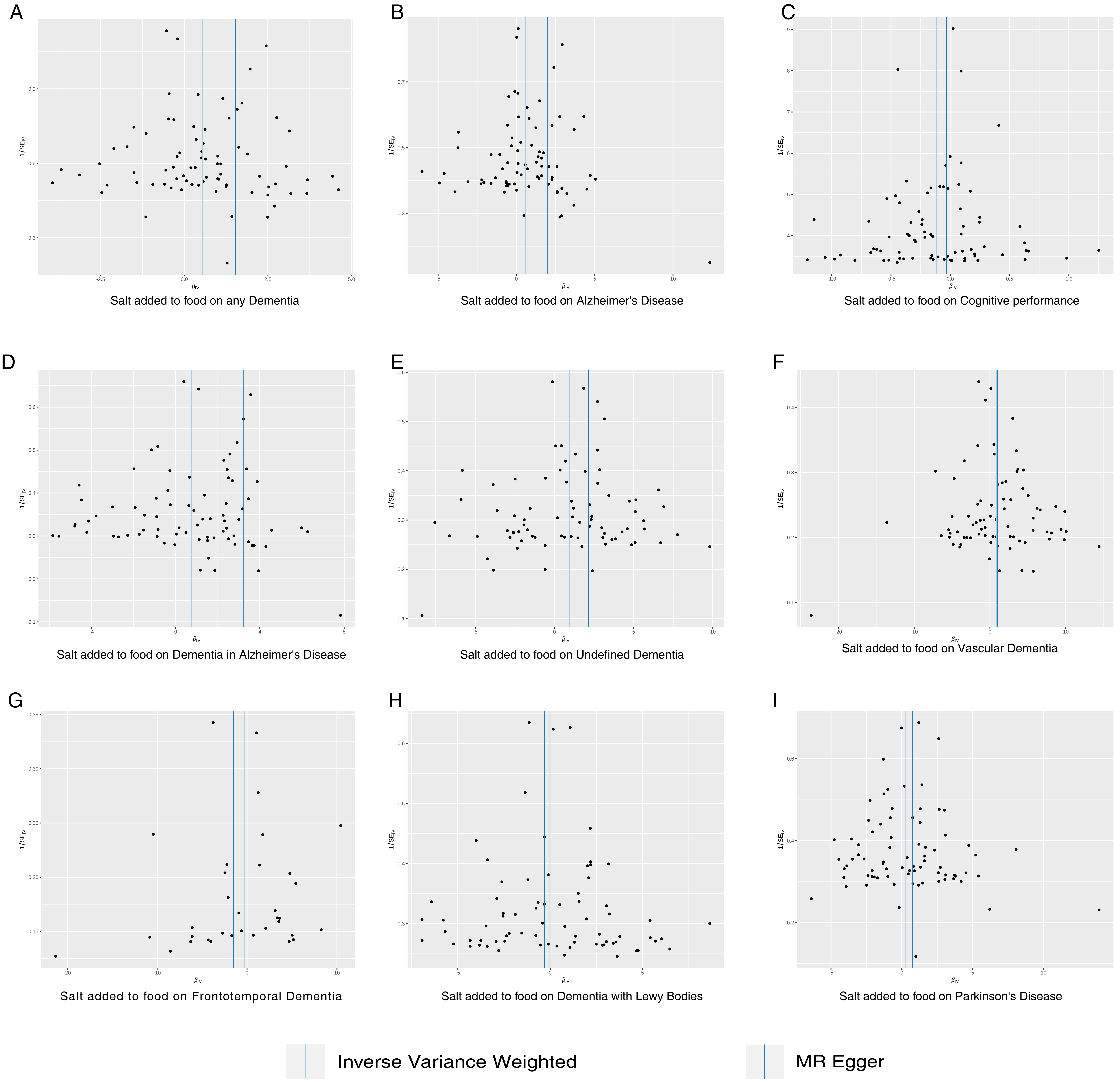
A
Salt added to food on any Dementia
B
Salt added to food on Alzheimer's Disease
C
Salt added to food on Cognitive performance
D
Salt added to food on Dementia in Alzheimer's Disease
E
Salt added to food on Undefined Dementia
F
Salt added to food on Vascular Dementia
G
Salt added to food on Frontotemporal Dementia
H
Salt added to food on Dementia with Lewy Bodies
I
Salt added to food on Parkinson's Disease
$1/SE_{IV}$
β_{IV}
Inverse Variance Weighted
MR Egger
